# Sarcopenia in head and neck cancer: A scoping review

**DOI:** 10.1371/journal.pone.0278135

**Published:** 2022-11-28

**Authors:** Nedeljko Jovanovic, Tricia Chinnery, Sarah A. Mattonen, David A. Palma, Philip C. Doyle, Julie A. Theurer

**Affiliations:** 1 Health and Rehabilitation Sciences, Western University, London, ON, Canada; 2 Department of Medical Biophysics, Schulich School of Medicine & Dentistry, Western University, London, ON, Canada; 3 London Health Sciences Centre, London, ON, Canada; 4 Lawson Health Research Institute, London, ON, Canada; 5 Division of Laryngology, Department of Otolaryngology–Head and Neck Surgery, Stanford University School of Medicine, Stanford, CA, United States of America; 6 Department of Otolaryngology–Head and Neck Surgery, London, ON, Canada; 7 School of Communication Sciences and Disorders, Elborn College, Western University, London, ON, Canada; Euro American University Center, BRAZIL

## Abstract

**Objectives:**

In those undergoing treatment for head and neck cancer (HNC), sarcopenia is a strong prognostic factor for outcomes and mortality. This review identified working definitions and methods used to objectively assess sarcopenia in HNC.

**Method:**

The scoping review was performed in accordance with Arksey and O’Malley’s five-stage methodology and the Joanna Briggs Institute guidelines.

**Information sources:**

Eligible studies were identified using MEDLINE, Embase, Scopus, Cochrane Library, and CINAHL databases.

**Study selection:**

Inclusion criteria represented studies of adult HNC patients in which sarcopenia was listed as an outcome, full-text articles written in English, and empirical research studies with a quantitative design.

**Data extraction:**

Eligible studies were assessed using a proprietary data extraction form. General information, article details and characteristics, and details related to the concept of the scoping review were extracted in an iterative process.

**Results:**

Seventy-six studies published internationally from 2016 to 2021 on sarcopenia in HNC were included. The majority were retrospective (n = 56; 74%) and the prevalence of sarcopenia ranged from 3.8% to 78.7%. Approximately two-thirds of studies used computed tomography (CT) to assess sarcopenia. Skeletal muscle index (SMI) at the third lumbar vertebra (L3) (n = 53; 70%) was the most prevalent metric used to identify sarcopenia, followed by SMI at the third cervical vertebra (C3) (n = 4; 5%).

**Conclusions:**

Currently, the most effective strategy to assess sarcopenia in HNC depends on several factors, including access to resources, patient and treatment characteristics, and the prognostic significance of outcomes used to represent sarcopenia. Skeletal muscle mass (SMM) measured at C3 may represent a practical, precise, and cost-effective biomarker for the detection of sarcopenia. However, combining SMM measurements at C3 with other sarcopenic parameters—including muscle strength and physical performance–may provide a more accurate risk profile for sarcopenia assessment and allow for a greater understanding of this condition in HNC.

## Introduction

Individuals undergoing treatment for head and neck cancer (HNC) are likely to experience significant weight loss [1] and substantial declines in physical activity, skeletal muscle mass (SMM), muscle strength, and overall physical performance [2, 3]. One condition characterized by these complications is termed sarcopenia. Sarcopenia is typically defined as an age-related muscle wasting condition that is associated with an increased likelihood of adverse outcomes such as falls, fractures, physical disability, and mortality [4]. Among older individuals between 60–70 years of age, the prevalence of sarcopenia is estimated to range between 5–13%, with a prevalence of up to 50% for individuals over the age of 80 years [5]. In HNC, the prevalence of sarcopenia has been reported to be between 35.5–54.5% [6]. Considering that approximately 35%-60% of all HNC patients may present with malnutrition and weight loss of greater than 10% [7], these numbers are not surprising. Moreover, organ sparing treatments such as radiotherapy (RT) or chemoradiotherapy (CRT) have the potential to exacerbate issues related to nutrition and weight management. For example, treatment-related toxicities including xerostomia, dysphagia, and oral mucositis, which are associated with reduced oral intake [8, 9], place the patient at an increased risk for malnutrition, weight loss, and consequently, sarcopenia.

In those undergoing HNC treatment, sarcopenia is associated with higher rates of chemotherapy toxicity and prolonged RT interruptions [10]. Researchers have also reported an association between pre-treatment sarcopenia and a modified diet, objective speech problems, xerostomia, swallowing-related QoL, and physician-rated dysphagia in those receiving definitive CRT [11, 12]. Diminished SMM measured by computed tomography (CT) imaging may also be associated with risk of prolonged feeding tube (FT) dependency and hospitalization, poor locoregional disease control, and increased rates of postoperative complications in HNC [13–15]. Given that HNC patients with sarcopenia exhibit greater degrees of systemic inflammation–a state suggested to be indicative of cancer aggressiveness and poor prognosis [16]–its association with poor outcomes in oncology and HNC is somewhat expected. In addition, the relationship between sarcopenia and survival outcomes are well-documented in the HNC literature. Evidence suggests that sarcopenia is a strong and negative prognostic factor for relapse-free survival, disease-free survival, progression-free survival, and overall survival [17, 18]. Grossberg et al. [14] reported that sarcopenia–as identified both before and after treatment–was associated with worse overall survival for HNC patients treated with CRT. Stone et al. [19] also found sarcopenia to be a significant negative predictor of both 2- and 5-year overall survival. Further, both a systematic review [20] and meta-analysis [21] concluded that the presence of sarcopenia is associated with a significant decrease in overall survival. Jung et al. [22] reported a three-fold increase in risk of overall recurrence, and even death in HNC patients diagnosed with sarcopenia, further emphasizing the importance of this condition as a prognostic factor.

The variability in prevalence data for sarcopenia obfuscates the actual prognostic significance of this condition. This variability may be attributed to the fact that sarcopenia is inconsistently defined across studies and consensus on the use of cut-off values to guide diagnosis and treatment is lacking [23]. Due to the challenges related to methods of measurement, including selection of variables to be measured and the most effective approach to evaluate the impact of therapeutic interventions [24], sarcopenia has been somewhat overlooked and undertreated in clinical practice [25]. As emphasized by the European Working Group on Sarcopenia in Older People (EWGSOP), “practitioners have ever-increasing possibilities for preventing, delaying, treating, and sometimes even reversing sarcopenia by way of early and effective interventions” [4, p.17]. Failure to identify and provide early intervention for individuals with sarcopenia may place them at risk for inferior treatment outcomes and cognitive impairment, impair their ability to perform activities of daily living, and contribute to financial burden, loss of independence, poor quality of life (QoL), and death [26–31].

The objective of this scoping review was to examine the extent, range and nature of the current research in the study of sarcopenia in HNC. This includes how sarcopenia was defined, the various methods of measurement for SMM, and how the classification of sarcopenia was decided. Knowledge gaps in the existing literature were also identified. A clearer understanding of how sarcopenia is currently assessed in HNC research is an important first step toward achieving consensus on its assessment in practice and research. Such consensus may serve to identify patients at risk for adverse outcomes in order to provide early, targeted interventions. Such efforts may allow for the indexing of sarcopenia as an accessible biomarker to identify patients who may benefit from proactive intervention, guiding clinical practice and facilitating improved outcomes. This information may also guide future research in this field.

## Methods

### Design and research questions

The methodology for this scoping review followed guidelines originally developed by Arksey and O’Malley [32] including the following five phases: (1) identifying the research question, (2) identifying relevant studies, (3) study selection, (4) charting the data, and (5) collating, summarizing, and reporting the results. The review was also guided by the Joanna Briggs Institute Manual for Evidence Synthesis [33]. In accordance with these frameworks, a quality assessment was not performed. The protocol for this scoping review can be accessed on The Open Science Framework (http://dx.doi.org/10.17605/OSF.IO/FD7WJ). The specific research questions used to guide the review were: (1) How is sarcopenia assessed in HNC patients? (2) What methods have been used to assess sarcopenia in HNC? (3) How is the term sarcopenia defined and what cut-off values are used to classify sarcopenia in those undergoing HNC treatment? (4) What are the knowledge gaps and/or directions for future research within publications based on the primary review question?

### Information sources and search strategy

To identify potentially relevant studies, searches were carried out using MEDLINE, Embase, Scopus, Cochrane Library, and CINAHL databases from the earliest available time until July 22, 2021. The search strategy was developed for MEDLINE and then adapted for other databases in consultation with an experienced Health Sciences Librarian. Team discussion regarding uncertainties or challenges related to methodology, inclusion and exclusion criteria, participant selection, and source characteristics further refined the search strategy to ensure an appropriate and thorough literature search. Backward citation searching (i.e., inspection of references cited within the sources of evidence recovered from the search) and forward citation searching (i.e., identification of articles that cite a source study using a citation index) were performed to identify additional articles [34] referring to sarcopenia in HNC. Reference lists of previous systematic reviews, meta-analyses, and scoping reviews were also manually searched to identify relevant studies. The MEDLINE search strategy is presented in **S1 Appendix**. The final search results were exported into Covidence (Veritas Health Innovation, Melbourne, Australia)–a web-based software supported through our institution’s library used to assist researchers in screening references and extracting data.

### Study selection

Peer-reviewed journal papers were considered for further review if they included adult HNC patients over the age of 18 years undergoing (C)RT and/or surgery and listed sarcopenia as an outcome. To capture a broad range of published evidence on the assessment of sarcopenia in HNC patients, full-text, empirical literature written in English that was performed with a quantitative research design was included. Sole consideration of studies with a quantitative methodology allowed for the inclusion of evidence that focused on radiologically defined sarcopenia as assessed with CT imaging.

As a first step, two reviewers independently screened each article title and abstract for eligibility based on the pre-specified inclusion and exclusion criteria using Covidence. During this initial review, articles for which inclusion eligibility was uncertain were maintained for further review (i.e., maintained in a pool of potentially eligible articles for full read). Next, backward and forward citation searching was performed to identify other relevant sources. Reviewers met at the beginning, middle, and final stage of the review process to address potential ambiguity and to ensure that selected abstracts were appropriate for full review. Finally, the resulting full-text version of all selected studies was retrieved and similarly screened. Disagreement between the reviewers was resolved through discussion or, if necessary, by a third reviewer. This review period took place over the course of four weeks.

### Data extraction

A data extraction form was developed by the research team to delineate which variables were to be extracted from included full-text articles (**S2 Appendix**). The following data were extracted and classified: general information (e.g., author[s], year of publication, and country of origin), article details and characteristics (e.g., aims/purpose, participant details, treatment type, methodology, and summary findings), and details/results from the source of evidence in relation to the concept of the scoping review (e.g., sarcopenia definition, sarcopenia cut-off value(s), measurement technique, timing of assessment, and knowledge gaps). Both reviewers independently charted the data. The results were discussed collaboratively, and the data-charting form was continuously updated in an iterative process. In accordance with the JBI scoping review protocol, the two researchers independently extracted data from the first five articles and then met to determine whether the approach to data extraction was consistent with the research question and purpose of the scoping review and that all relevant information was extracted [33]. Any changes to the form resulting from the pilot process were recorded.

## Results

**Fig 1:**
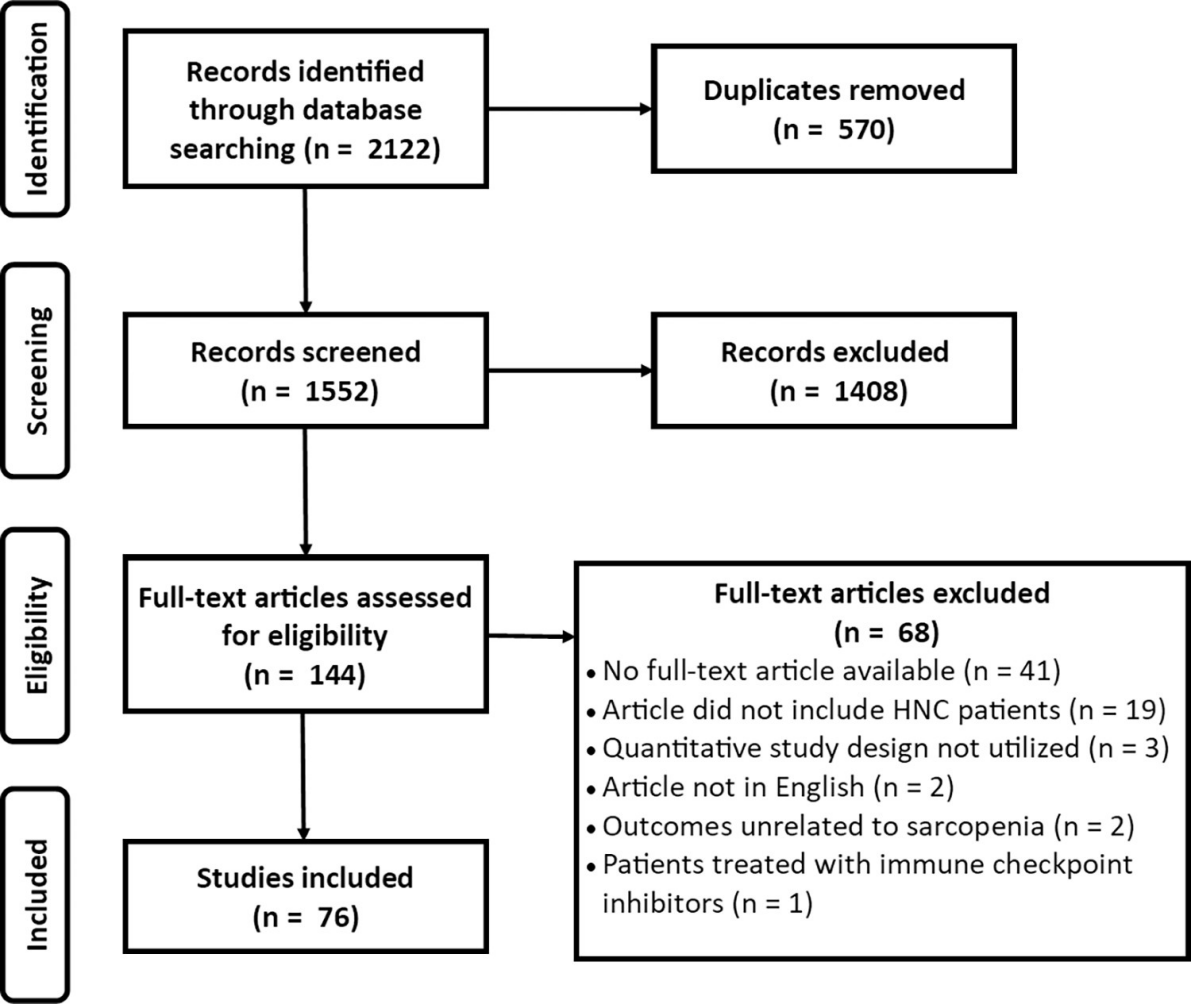
PRISMA flow chart for the scoping review process.

The current review was performed to examine the definition, measurement, and classification of sarcopenia in HNC. A total of 1552 peer-reviewed articles were retrieved from all databases after duplicates were removed. Upon initial screening based on titles and abstracts, 144 studies met the inclusion criteria. The full-text version of these articles were evaluated for eligibility and 68 were excluded for the following reasons: no full-text article available (n = 41), the article included patients with other cancer sites (n = 19), a quantitative design was not utilized (n = 3), the article was not written in English (n = 2), the outcomes were unrelated to sarcopenia (n = 2), and patients were treated with immune checkpoint inhibitors instead of (C)RT therapy and/or surgery (n = 1). Thus, 76 studies were included in this review. The study selection process is detailed using a PRISMA flow chart in **Fig 1**.

### Study details and characteristics

**Fig 2:**
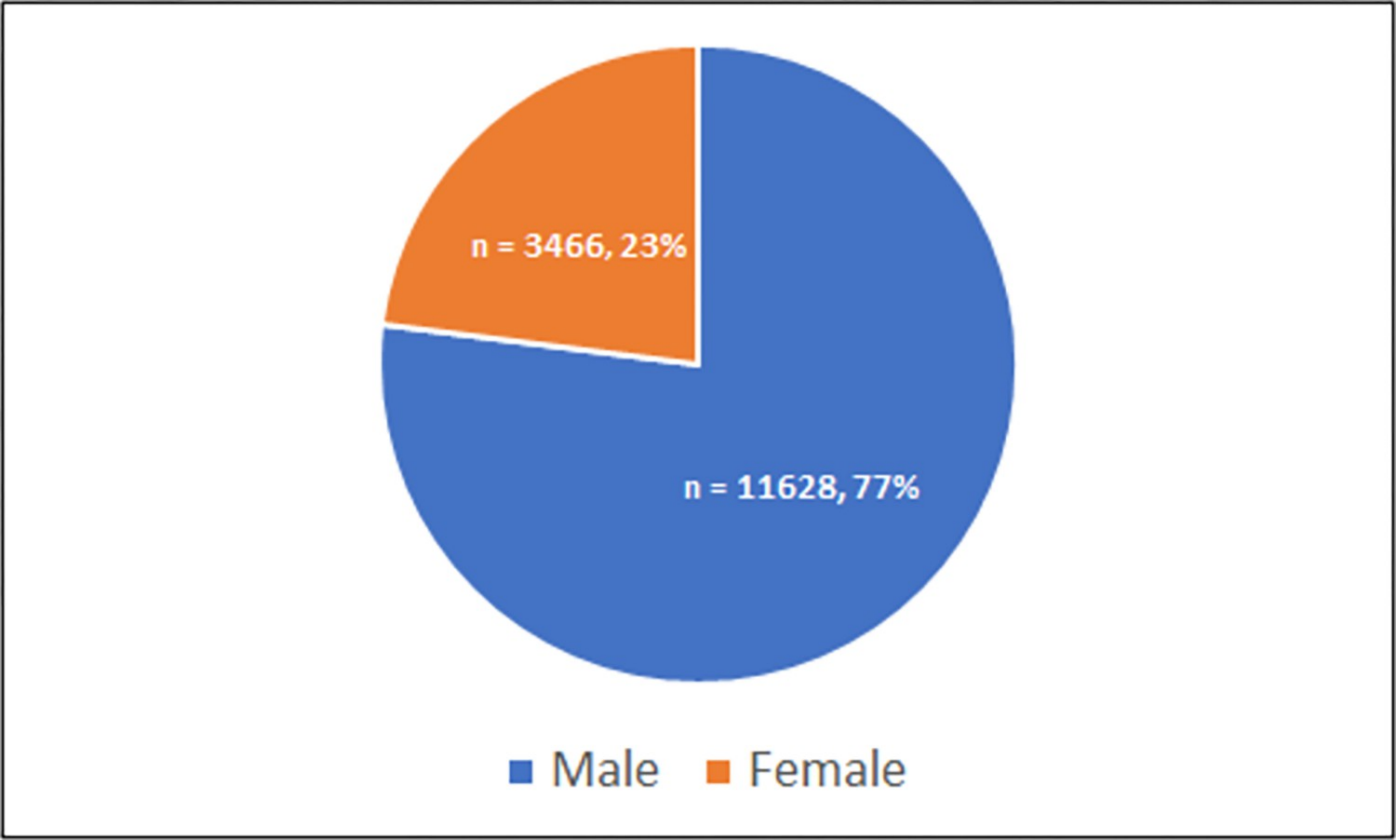
Participant details (sex).

**Fig 3:**
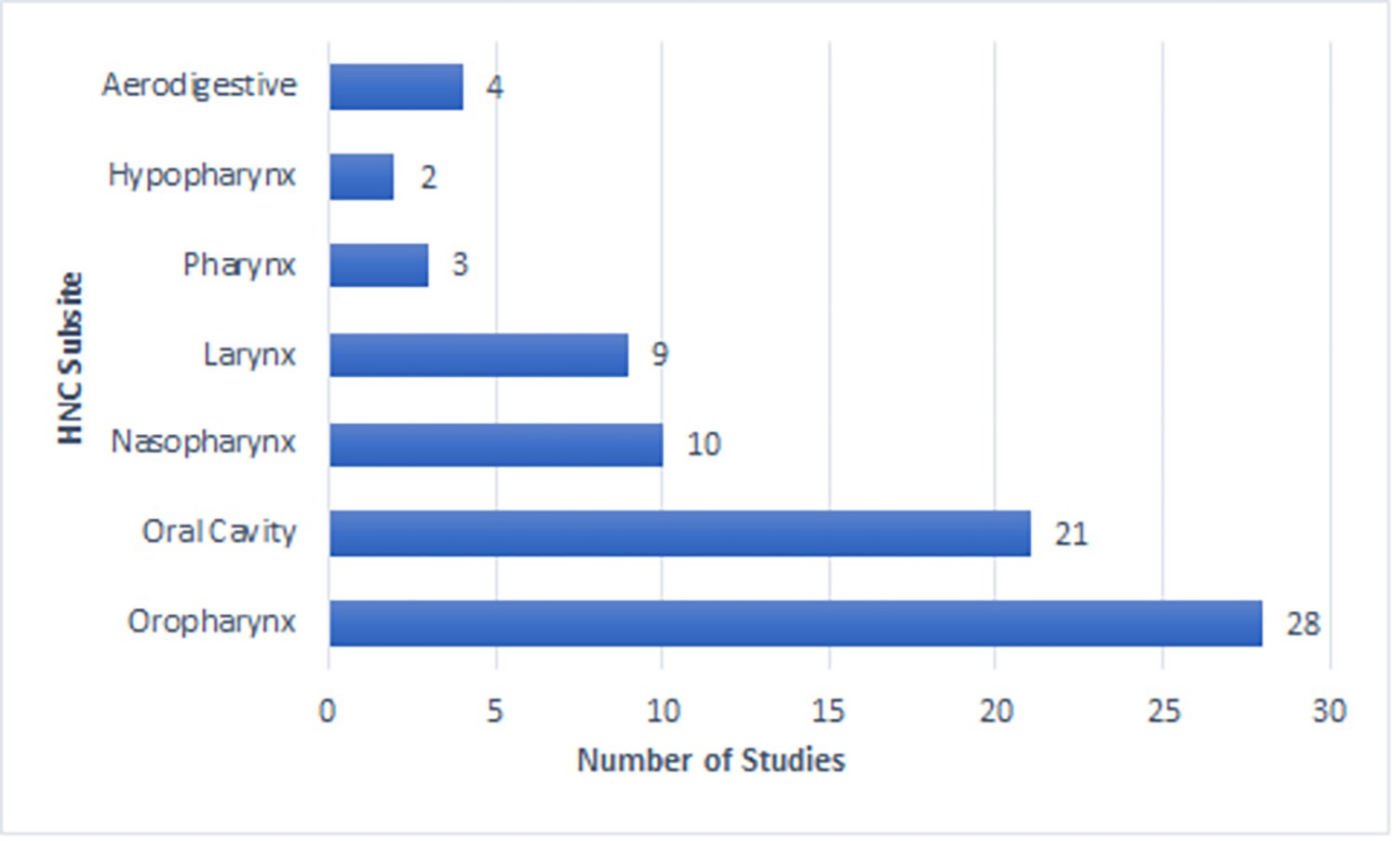
Participant details (tumour subsites).

**Fig 4:**
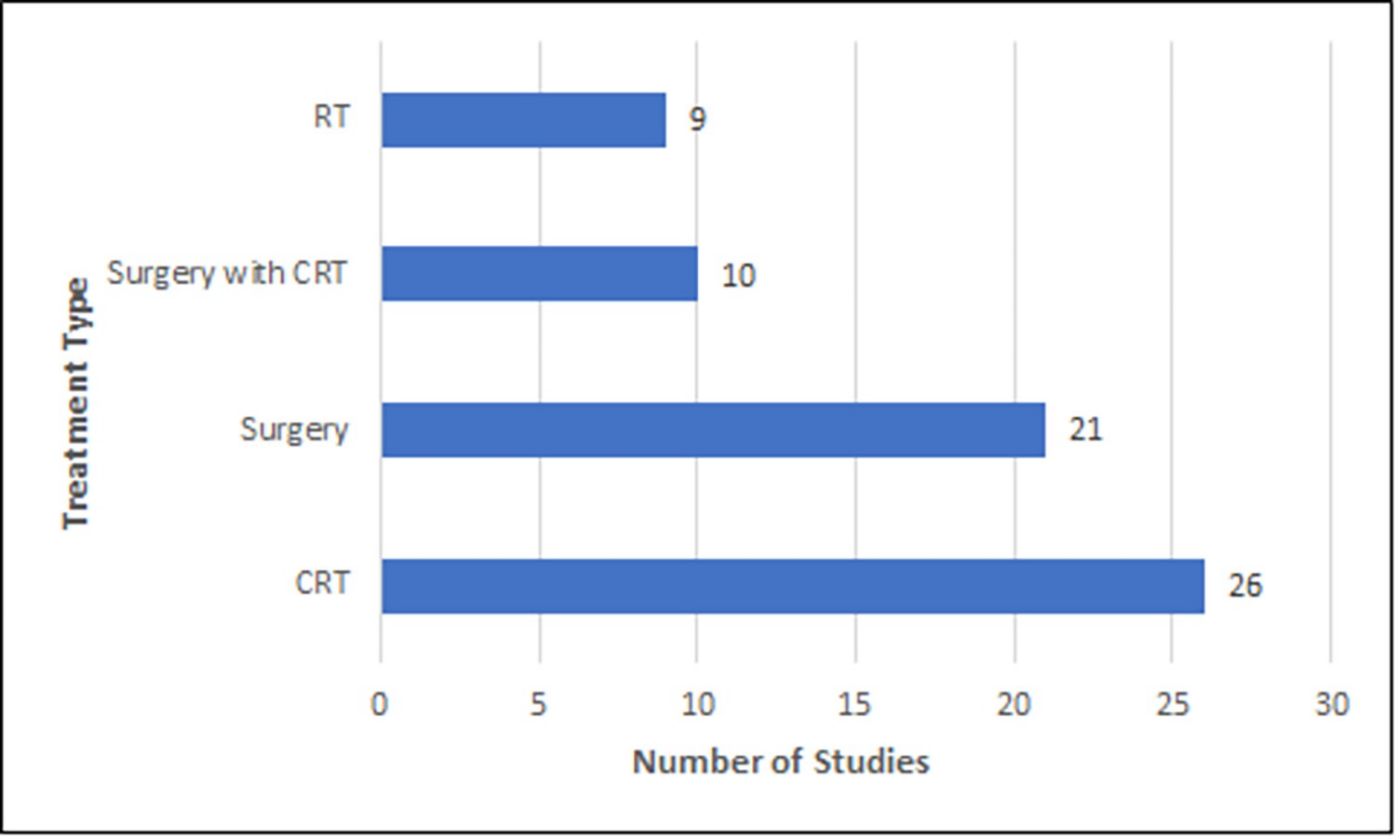
Participant details (treatment type).

The 76 articles included in this scoping review represent international research published from 2016 to 2021. A total of 14 countries were represented including Netherlands (18), United States of America (14), Japan (11), China (7), Taiwan (7), Republic of Korea (5), Canada (3), Turkey (3), France (2), Italy (2), Australia (1), Brazil (1), Finland (1), and India (1). The sample size for included studies ranged from 19 to 1767 participants with the average age ranging from 45 years to 81.73 years. The majority of participants represented were male (**Fig 2**) and the most common tumour subsite was the oropharynx followed by the oral cavity (**Fig 3**). Four articles (5%) used the term “aerodigestive” to specify subsite, which included a combination of the oral cavity, oropharynx, hypopharynx, and/or larynx. Most tumours were primary (n = 67; 88%) and stage IV (n = 39; 51%). CRT and surgery were the most common treatment modalities across the studies included in this scoping review (**Fig 4)**.

The majority of included articles utilized a retrospective study design (n = 56; 74%). Eighteen studies were prospective in nature (24%), including one randomized controlled trial. Sixty-two studies (82%) were aimed at determining the prognostic impact of sarcopenia as the primary objective, eight (11%) investigated the association between specific outcomes/interventions and sarcopenia, five (7%) were designed to compare body composition measurements before and after treatment, and four (5%) were designed to determine the prevalence of sarcopenia. The remaining studies explored different measurement techniques for assessing sarcopenia in HNC patients.

Among the included studies, survival was the most prevalent primary outcome measure and was investigated as the dependent variable in association with sarcopenia in 68 publications. Complications and treatment-related toxicities were the second most common primary outcomes (n = 29), followed by body composition (n = 13), frailty (n = 4), interruptions to treatment (n = 3), and nutrition-related measurements (n = 3). Sarcopenia was found to be a significant predictor of outcomes in 62 studies (82%). Across studies, the prevalence of sarcopenia ranged from 3.8% to 78.7%; 19 articles (25%) did not provide prevalence estimates. In addition, prevalence was not determined in 14 studies (18%) that interpreted SMM as a continuous variable. Characteristics and details of articles included are summarized in **S3 Appendix**.

### Definition and operational outcomes

**Fig 5:**
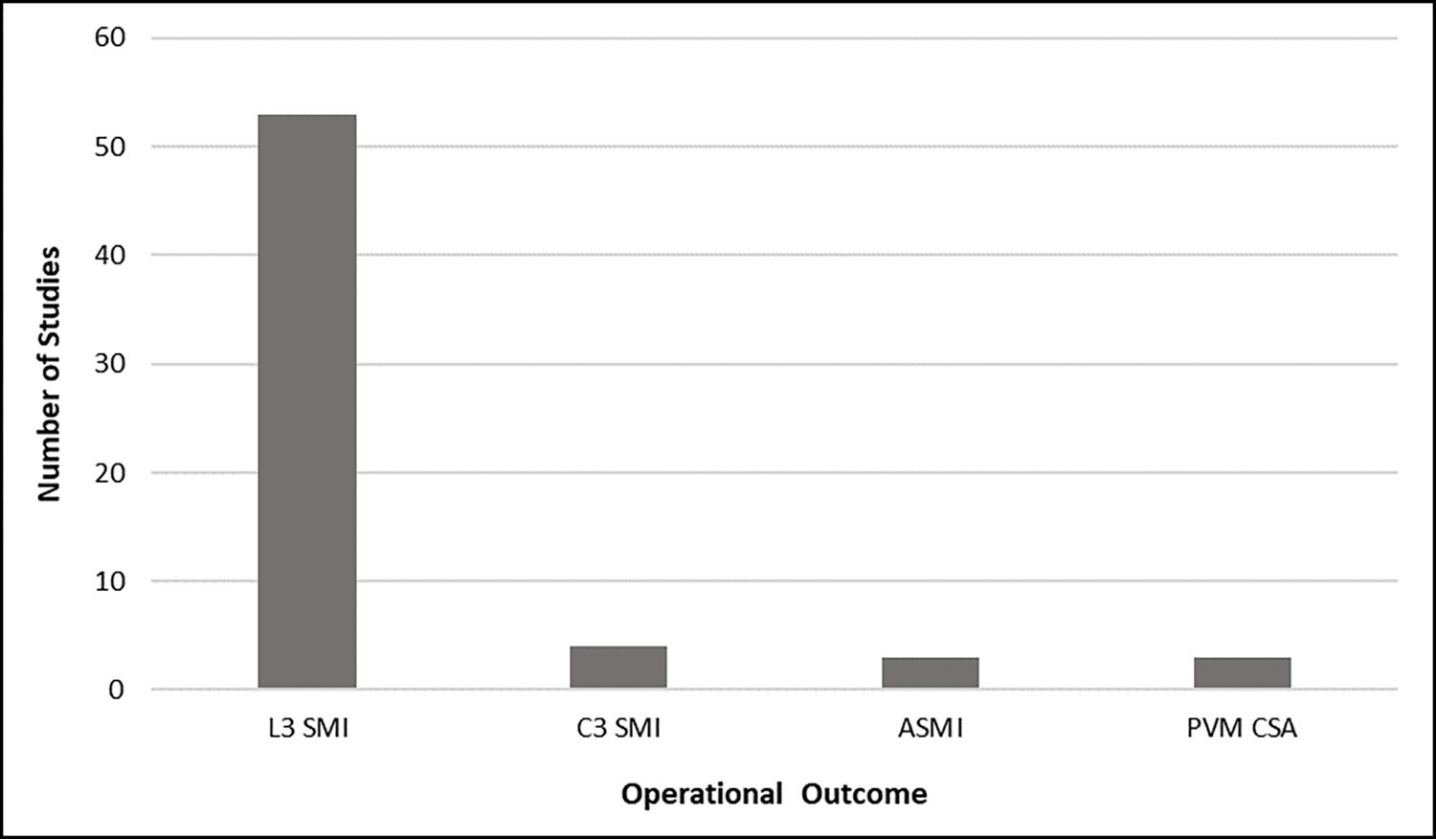
Prevalence of outcomes used to represent sarcopenia.

In 28 studies (37%), sarcopenia was defined as low SMM. Sarcopenia also was defined as low SMM and low function in four separate articles, and as low SMM, low strength/function and affected physical performance in 10 articles. The definition included a specific reference to “progressive and generalized” loss in 10 studies, and “age-related” in four articles. Muscle quantity was the primary variable used to assess sarcopenia and was measured in every study included in this scoping review. While the majority of articles (n = 67; 88%) normalized measurements for patient height and used skeletal muscle index (SMI) as the primary outcome, 10 studies (13%) used the cross-sectional area (CSA) of musculature alone to quantify SMM. The most prevalent outcome used as a marker of SMM quantity was third lumbar vertebra (L3) SMI, which was used in 53 studies (70%). Third cervical vertebra (C3) SMI was the second most common operational outcome (n = 4; 5%). Appendicular SMI and paravertebral muscle (PVM) CSA were used as the primary outcome in three studies (4%), and L3 CSA was measured in two articles (3%) included in this review (**Fig 5)**. Twenty-one of 29 articles (72.4%) that used CT imaging of the neck to determine SMM were found to have applied the algorithm developed by Swartz and colleagues (2016) to convert measurements at C3 to L3 SMI. In seven studies that measured muscle strength, a handheld dynamometer was consistently used to measure handgrip strength. Physical performance was measured using a stopwatch to measure the Timed Up and Go (TUG) test (n = 2), and gait speed (n = 3) in five articles. Only one study used the self-reported strength, assistance with walking, rising from a chair, climbing stairs, and falls (SARC-F) questionnaire to assess sarcopenia.

### Measurement technique

Nearly two-thirds of studies (64%) utilized CT imaging to measure SMM (n = 49), with positron emission tomography (PET)-CT identified as the primary instrument in 25 studies (33%). Other less common measurement instruments included magnetic resonance imaging (MRI), dual-energy x-ray absorptiometry (DEXA), bio-electrical impedance analysis (BIA), and b-mode ultrasonography. The most common location of SMM measurement was at L3 (n = 37; 49%). Of the studies that conducted SMM measures at L3, 17 delineated abdominal wall musculature (i.e., transversus abdominus, external and internal obliques, rectus abdominus, psoas, erector spinae, and the quadratus lumborum), eight described delineating all muscles at L3 (i.e., the entire L3 vertebral arch and transverse process), and one study quantified the right and left psoas muscles to determine SMM quantity. C3 was the second most common location for SMM measurement (n = 29; 38%). Twenty-four of the articles using the C3 location delineated both the PVM and sternocleidomastoid (SCM) muscles to obtain a measure for SMM quantity; the PVM and SCM were delineated separately in three articles. Full body (n = 5; 7%) and appendicular (n = 3;4%) SMM were also used to assess sarcopenia.

To delineate muscles and provide an estimate for SMM quantity, SliceOmatic (TomoVision, Montreal, Canada) (n = 20; 26%) and ImageJ (National Institutes of Health and the Laboratory for Optical and Computational Instrumentation, University of Wisconsin, Wisconsin, USA) (n = 7; 9%) software were commonly used. Other computer software used for this purpose included Volumetool (University Medical Center Utrecht, The Netherlands) (n = 3; 4%), Pinnacle (Philips Radiation Oncology Systems, Andover, Massachusetts, USA) (n = 3; 4%), Aquarius Workstation (TeraRecon, California, USA) (n = 3; 4%), InBody (Biospace, Seoul, Korea) (n = 3; 4%), and Monaco TPS (Elekta, United Kingdom) (n = 3; 4%). Measurements were most often obtained manually by a radiation oncologist (n = 15; 20%). Twenty studies (26%) noted that either a researcher, observer, or examiner were involved in the measurement of SMM. One study utilized a deep learning segmentation algorithm to delineate muscles automatically. In 38 articles (50%), detailed information regarding the assessor was not provided. Assessors were blinded to patient outcomes in nine studies (12%) and only seven studies (9%) included reliability analysis with respect to the assessment of sarcopenia.

### Cut-off values

The present scoping review revealed that 33 different cut-off (i.e., threshold) values have been used to diagnose sarcopenia in HNC patients. Thirty-five studies (46%) applied gender-specific cut-offs, with four articles (5%) using different threshold values based on the individual’s body mass index (BMI) (i.e., different threshold applied to those who are underweight versus overweight). Fourteen articles (18%) measured and analysed SMM as a continuous variable and, therefore, did not apply cut-off values in their assessments. Recognizing the variation in sarcopenia assessment, most authors provided an explanation for their selection of specific threshold values. Thirty-six studies (47%) applied a cut-off based on previously published values, with 11 and 10 of these articles citing work by Prado et al. [35] and Wendrich et al. [36], respectively. Thirteen studies (17%) used receiver operating characteristic (ROC) curve analysis to determine a cut-off value that was specific to their sample and outcomes of interest. Other threshold values were based on the lowest log-likelihood values (n = 3), the lowest gender-specific quartile (n = 2), the EWGSOP definition (n = 1), and optimal stratification techniques (n = 1).

### Timing of assessment

Within the body of literature included, sarcopenia was most often investigated as a predictor of outcomes. This is reflected in the large proportion of studies that measured SMM prior to treatment (n = 75, 99%). Despite the frequent assessment of sarcopenia pre-treatment, the time elapsed between assessment and start of treatment greatly varied. Assessment occurred from time of diagnosis, during tumour staging, and the moment of admission, all the way up to the outset/beginning of treatment. Thirty-two of these studies failed to provide detailed information regarding timing of assessment, stating only that sarcopenia was assessed “prior to treatment”. Sarcopenia was assessed throughout the course of treatment in four studies (5%). Post-treatment sarcopenia assessment was reported in 21 studies (28%), ranging from immediately at the end of treatment to within one year after treatment. Four of these studies lacked temporal specificity, describing sarcopenia assessment as only occurring “after treatment”.

## Discussion

### Study details and characteristics

The current scoping review reports findings from 76 studies identified through a systematic literature search and published over a 6-year period (2016–2021) on the study of sarcopenia in HNC. Despite the growing body of literature in this field of research, discrepancies related to the definition, measurement, and assessment of this muscle-wasting condition present challenges in the interpretation of results and their application to patient care. Such discrepancies are reflected in the large range of reported prevalence of sarcopenia among included studies. The significant variability in prevalence may be attributed to inconsistency in applied cut-off values, which muscles are measured and at which position, and the specific outcome(s) used in the operational definition of sarcopenia. This may be considered as a substantial limitation within the literature and may contribute to difficulties in studying and in turn rectifying the issue of SMM loss in the HNC population.

Despite the inconsistencies related to its definition and assessment, sarcopenia is consistently reported to be a *significant predictor of adverse outcomes* in HNC. Sixty-two studies (82%) found sarcopenia to be a *significant prognostic* variable (**S3 Appendix**), providing a degree of confidence that the SMM quantity on its own may have clinical value. Consistent with these findings, two recent systematic reviews and meta-analyses demonstrated that based on SMM alone, sarcopenia had a significant and negative impact on survival outcomes in HNC [20, 21]. However, due to the variability in the definition and assessment of sarcopenia, the conclusions made within these sources of evidence are difficult to interpret. Further, despite the abundance of evidence demonstrating the prognostic implications of sarcopenia and low SMM, the scarcity of research on its impact on functional, psychosocial, and QoL outcomes can be considered a significant gap in this area of research. To determine the full extent to which sarcopenia may impact individuals with HNC, a more comprehensive analysis is needed with regard to its impact on multiple outcomes.

### Linguistic and operational definition of sarcopenia

Our review highlighted substantial heterogeneity with respect to the linguistic and operational definitions of sarcopenia. In its most recent definition, the EWGSOP noted that low muscle strength–identified as the most reliable measure of muscle function–should be used as the primary variable for sarcopenia assessment [4]. Sarcopenia is probable when low muscle strength is detected, confirmed by the presence of low SMM, and considered severe when low physical performance is detected as well [4]. Providing a well-rounded and accurate description of sarcopenia, therefore, requires that all three parameters be included in the assessment of this muscle-wasting condition. However, in the majority of articles included in this scoping review, the primary parameter used to define and assess sarcopenia was low SMM quantity. Muscle strength has seldom been addressed, with severity (i.e., through the detection of low physical performance) rarely considered as well. This may be explained by the abundance of retrospective studies on sarcopenia in HNC. Because CT imaging of the head and neck is routinely performed in the context of HNC treatment, SMM measurements will often be available even with retrospective analyses. On the other hand, muscle function or muscle strength is often not assessed routinely, limiting its application in retrospective work and offering an explanation for the heterogeneity in the operational definition of sarcopenia in the present review. The fact that SMM measurements alone were primarily used to assess sarcopenia may overestimate its true prevalence and, moreover, the impact of this condition. Thus, assessment based on all three components of sarcopenia (i.e., muscle strength, muscle mass, and physical performance) is warranted in order to improve consistency and accuracy within sarcopenia research and to facilitate its use as a prognostic factor in clinical practice.

An important consideration with respect to these findings is the potential advantage of using a multifactorial definition. Because the comprehensive definition of sarcopenia is narrow, assessing all three parameters outlined in the EWGSOP definition may help to identify an even more homogeneous, at-risk group of patients that would especially benefit from early intervention. For example, although Ganju and colleagues [10] found that sarcopenia was not a significant prognostic factor in p16-positive HNC patients, they only used reduced SMM as the criteria for assessing sarcopenia. In examining the association between sarcopenia and frailty, Meerkerk et al. [37] used reduced handgrip strength in addition to the loss of SMM to assess sarcopenia. The prevalence of sarcopenia was approximately 14%, even though 61% of patients were reported to have low SMM. The prevalence of sarcopenia in a study that included all three measures was found to be even lower at 3.8% [38]. It is apparent that a well-rounded assessment of sarcopenia using all three parameters (i.e., muscle quantity, muscle strength, and physical performance) would provide a more accurate risk profile and allow for a greater understanding of the true impact of this condition.

On the other hand, one unintended consequence associated with the application of this comprehensive definition may be that patients who are at risk but do not meet the full criteria for sarcopenia diagnosis (i.e., they only experience one of the three parameters) may be missed. Moreover, using more metrics in the assessment of sarcopenia may increase the difficulty of obtaining measurements; measuring SMM using CT imaging alone is simply and does not subject the patient to additional testing and the associated burden. Considering that sarcopenia based solely on the loss of SMM is present in 35.5–54.5% of patients with HNC and is related to adverse health outcomes [14, 39], SMM alone appears to hold clinical and prognostic value. In future studies, researchers should aim to use all three measures, both in combination and independently, to investigate potential differences in prevalence and to determine which parameters lead to more significant changes in patient outcomes.

The need for a more consistent approach in the assessment of sarcopenia is also evident given the inconsistencies between linguistic and operational definitions. For example, in some articles, a full and comprehensive definition of sarcopenia outlining all three parameters described by the EWGSOP is presented in the introduction. However, only SMM quantity is used to determine the prevalence and impact of sarcopenia. Although muscle quantity was measured in every article, muscle strength and physical performance were only measured in seven and five studies, respectively. Given that a comprehensive linguistic definition incorporating all three parameters was provided in 13 articles, it is evident that even when sarcopenia is accurately defined, comprehensive assessment does not consistently occur. Thus, it appears as though the primary driver for the assessment of sarcopenia in HNC lies in the operational definition, rather than the linguistic.

### Method of measurement

Given the heterogeneity in methods used to measure and characterize sarcopenia in HNC, selecting the most appropriate approach may be challenging. The findings of this scoping review suggest that the “ideal” choice may be contextual and dependent on several elements, namely resources, patient and treatment characteristics, and the accuracy of selected outcomes. The first and perhaps most important factor in determining whether a person has sarcopenia is symptomology. A patient may report experiencing signs or symptoms of sarcopenia such as fatigue, falling, difficulty standing from a seated position, weight loss, and/or muscle wasting, prompting the need for further testing to confirm its presence [40]. To elicit self-reported information from patients in order to identify these potential symptoms, the EWGSOP recommends a 5-item questionnaire known as the SARC-F. Proven to be a valid, consistent, and effective tool, the SARC-F questionnaire is recommended as an inexpensive and convenient method to identify individuals at risk for sarcopenia [4]. Although the SARC-F is a valuable tool for identifying risk based on one’s perception of their experience with personally relevant adverse outcomes (i.e., limitations in walking ability, standing out of a seated position, strength, climbing stairs, and experiences with falling) [31], it is limited by its self-reported nature, subjectivity, and low sensitivity [41]. In this scoping review, only one article used the SARC-F questionnaire to assess sarcopenia. Thus, a more objective, formal instrument may be needed to accurately diagnose sarcopenia in both research and clinical settings. Given that symptomology is an important factor in sarcopenia assessment, use of the SARC-F in combination with other objective measures may facilitate a more comprehensive and accurate method of assessment.

Currently, radiologically-defined sarcopenia is a term used to describe a technique that identifies sarcopenia through use of CT and/or MRI [42]; our review revealed that the majority of articles assessed sarcopenia using these two methods. The advantages of using CT and MRI for body composition analysis are well-known, as they are considered to be the “gold standard” for non-invasive measurement of muscle quantity [43]. For individuals diagnosed with cancer, CT scans are commonly used to acquire images for tumour staging and to monitor response to treatment. Accordingly, such imaging provides a convenient and precise method for assessing sarcopenia in HNC. Historically, SMM has been measured on abdominal CT scans at the level of L3 due to the accuracy and strong correlation of the measurement with total body SMM [44, 45]. Although CT scans at L3 are routinely available in some oncology populations (e.g., abdominal oncology patients) [46], performing abdominal imaging is not standard practice in diagnostic work-up in HNC. Accordingly, Grossberg and colleagues [14] reported that 93% of individuals receiving HNC treatment lack abdominal imaging with CT. However, recent methods supporting the measurement of SMM in HNC using neck imaging at C3 have been described. Swartz et al. [47] reported a strong correlation between the CSA of skeletal muscle at L3 and C3 and subsequently developed an algorithm for conversion. Because CT scans at C3 are routinely available in the diagnostic work-up for those undergoing HNC treatment [47], SMM can be measured without requiring additional imaging and patient burden. This trend is reflected in the findings of our scoping review, as 21 of 29 articles (72.4%) which used CT imaging of the neck to determine SMM were found to have applied the algorithm developed by Swartz et al. [47]. Moreover, all studies selected for inclusion were published after 2016 –the same year Swartz and colleagues developed the algorithm allowing for SMM measurement using CT scans of the head and neck at C3. This development explains the time frame of included studies (i.e., from 2016 to present), highlighting the utility and applicability of the algorithm and subsequent ease of SMM measurement in HNC.

#### Lumbar

Commonly reported as total body SMM, estimations for muscle quantity can be made through a variety of techniques that adjust for one’s BMI or height to achieve a more accurate and relevant measurement [48]. Considering the variation in SMM between individuals with similar skeletal muscle area but differences in height, it may be considered problematic to assess sarcopenia using only the CSA of skeletal musculature at any given location. Thus, to accurately diagnose sarcopenia, the use of a normalized measure of CSA incorporating the height of each individual is recommended. One such measure is SMI. Normalizing the CSA of muscles obtained on axial CT imaging for patient height to determine SMI is considered the international gold standard for body composition analysis in the quantification of SMM [49]. This is reflected in our scoping review by the tendency of researchers to favour SMI measurements in their assessment of SMM and sarcopenia and, in fact, the majority of articles did use SMI as the primary outcome.

SMI at the level of the L3 was most commonly used to represent sarcopenia. This is not unexpected given its strong correlation with whole-body SMM [48]. However, one potential concern regarding the use of L3 SMI as an indicator of sarcopenia is the process required to obtain lumbar imaging. In the present scoping review, nearly one third of included studies used PET-CT to determine L3 SMI. Because whole-body imaging (i.e., PET-CT) is primarily performed in HNC patients deemed to be of high risk for distant metastases or in those with advanced-stage disease [50], this method may introduce a substantial risk of selection bias, thus weakening the internal validity of findings and limiting their external applicability. Further, delineation of muscles at L3 may result in the overestimation of SMM measurement due to the complexity of L3 musculature and the potential need for third-party programs to provide accurate measurements [47]; applying this algorithm to convert C3 SMI to L3 SMI (without the need for PET-CT) can be a reliable alternative to using full-body imaging. However, the practice of deriving lumbar SMI estimations from C3 measurements is not without limitations as these estimations may be susceptible to calculation bias and differ from true L3 measurements [51].

#### Head and neck

The use of head and neck CT scans to measure muscle area at C3 was the second most common strategy for sarcopenia assessment within the present studies. This finding was expected given that CT imaging at the level of C3 is a part of the routine imaging protocol for those undergoing HNC treatment [52]. CT imaging of the head and neck may also limit radiation exposure compared to the full-body imaging required for lumbar measurements, thereby resulting in less patient burden [53]. SMM measured at C3 may be indicative of physical activity and nutritional status, emphasising the importance of early detection to implement corrective strategies. In addition, Ufuk et al. [54] found that muscle mass quantity measured directly at C3 (i.e., C3 SMI) was best able to discriminate sarcopenia in male HNC patients. Skeletal muscle measured at the level of C3 also shows excellent inter- and intra-observer agreement, suggesting that C3 SMI is both reliable and reproducible [6].

In addition to being a robust indicator of sarcopenia and having prognostic value for predicting duration of feeding tube (FT) use [15] and survival among patients with HNC [51], C3 SMI also has the advantage of being measured directly from conventional head and neck CT images. While CT-measured markers of sarcopenia are relatively straightforward to obtain, most other objective measurements require 5–10 minutes per patient which may be difficult to accommodate in real-time clinical practice [37]. Given that C3 SMI is easily identifiable on CT imaging, shows a strong and significant correlation with L3 SMI, and has proven prognostic value [22, 54], the conversion of these measurements to L3 may be unnecessary. C3 SMI could be used in order to limit the time commitment required to measure SMM and the potential for calculation bias when converting C3 measurements to L3 SMI. Doing so may facilitate accurate, real-time SMM measurement and allow for early therapeutic intervention to reduce the severity of sarcopenia and its complications. Still, SMM measurement at C3 is a relatively new concept with few studies available. More evidence is needed before C3 SMI can be used as a reliable method for body composition analysis and sarcopenia assessment in HNC.

While SMM measurements at C3 offer a cost-effective, feasible, and accurate alternative to L3 measurement in HNC patients, muscle delineation may be hindered due to metastatic cervical lymphadenopathy or previous neck dissection in locally advanced or recurrent HNC [47]. In evaluating the robustness of varying CSA measurements at C3, Bril et al. [52] concluded that interobserver agreement for the CSA of PVM muscles is most uniform; the highest level of variation was observed in SCM CSA measurements. One potential explanation for this observation is that the identification of muscles on head and neck CT imaging may be impaired by the presence of lymph node metastases [47]. Because lymph node stations are positioned closely around these muscles [55], the SCMs in particular are at high risk for lymph node invasion that can interfere with assessment. Swartz et al. [47] reported that lymph node metastases was responsible for the impaired measurement of SCM muscles in 11% of individuals with HNC, while PVM muscle measurement was possible in almost every patient. Moreover, Ufuk et al. [54] found that SCM measurement was impaired due to lymphadenopathies in 6.9% of HNC patients. In the context of real-world clinical care, approximately 57% of those undergoing HNC treatment present with lymph node metastasis [56]. Consequently, researchers should exercise caution when utilizing SCM measurements to assess sarcopenia if their study sample includes a high percentage of lymph node positive patients. To address the potential for impaired measurement related to lymph node metastases, it has been suggested that doubling the CSA of the SCM muscle that can be measured without interference is a reliable, equally predictive alternative. This limitation can also be minimized by excluding the SCM altogether from CSA calculations, given that the CSA of PVM alone correlates well with CSA at L3 [47].

#### Appendicular

Appendicular SMI (ASMI), or the sum of muscle mass of the upper and lower extremities adjusted for height, was also used as a marker of muscle quantity in three articles. Yeh et al. [57] found that the impact of CRT, with respect to lean mass, was greater in the peripheral extremities, indicating that sarcopenia assessed using ASMI measurements is associated with a lower tolerance to treatment. In addition, when compared to body weight, BMI, total lean mass and total fat mass, pre-treatment ASMI was reported to be the only prognostic factor capable of predicting 2-year recurrence-free survival [57]. These findings are supported in the literature with researchers suggesting that patients with low ASMI have low physical functioning [58] and are more likely to experience complications such as pneumonia or infection [59]. Consequently, it may become more difficult for patients to tolerate treatment. Researchers postulate that the strength of ASMI as a prognostic variable may be due to its potential association with full-body energy reservoir status. For example, in patients with a high ASMI, loss of muscle mass was observed throughout the entire body, while those with a low ASMI only had significant loss of lean mass within the appendicular skeletal muscle that was not observed in other regions of the body [57].

Moreover, a high ASMI is associated with a well-functioning mitochondrial ATP synthesis cycle, thereby allowing the entire body to exchange lean mass for much-needed energy during times of muscle wasting, such as during CRT. Because skeletal muscle functions as a metabolic organ and has the ability to produce energy through mitochondrial ATP synthesis [60], and given that HNC patients commonly experience significant energy deficits related to impaired dietary intake and metabolic derangement, the use of ASMI as a marker of sarcopenia may be appropriate in studies that investigate patients receiving CRT; it may be especially applicable in situations where oral intake is impaired and significant weight loss occurs. Nevertheless, the measurement of appendicular muscle mass requires access to modalities such as DEXA which can involve radiation exposure and are not practical or routinely performed in HNC care.

### Masticatory

Sarcopenia assessment is often centered around the degree to which measurements can accurately characterize whole body SMM. Because the term ‘generalized’ is considered a primary component of sarcopenia-associated SMM loss, the importance placed on whole-body SMM is warranted. Nevertheless, in HNC, it is the swallowing musculature that is often negatively impacted by treatment. Changes in the masticatory muscles can act as a reflection of swallowing function and nutritional status [61], with research also suggesting that these measurements are valid markers of sarcopenia in patients with trauma [62]. Masticatory function is not only vital for mechanical breakdown of foods, but associations with handgrip strength, walking speed, and physical fitness—some of the primary characteristics of sarcopenia according to the EWGSOP–have also been documented [63, 64]. Accordingly, these measurements have the potential to be used as an alternative to L3 imaging in sarcopenia assessment.

Research also has shown that the size of the primary masticatory muscles has a significant association with systemic nutritional biomarkers, even more so than the paraspinal muscles which are frequently used as measures of sarcopenia when lumbar CT imaging is used for assessment [65]. Although only one study used masticatory SMI (MSMI) to assess sarcopenia in the current scoping review [66], the findings have significant implications for the application of this measurement in HNC. In their assessment of sarcopenia using MSMI, Chang et al. [66] reported a significant relationship between sarcopenia and male sex. Moreover, they also noted that a potential advantage to using MSMI is that there is minimal opportunity for interference in this anatomical area for most cancers of the head and neck. Chang and colleagues [66] noted that masticatory muscles could be clearly identified in CT scans of the head and neck and that neither primary tumour nor lymph node invasion was observed. Moreover, MSMI measurements obtained through manual delineation and threshold selection are consistent and may be less influenced by different measures [66]. Thus, MSMI measurement may be feasible in most, if not all, HNC patients. Nonetheless, the application of the masseter muscle in the assessment of sarcopenia presents an inherent limitation, as its measurement may be dependent on external factors such as dental status and craniofacial structure [67].

### Psoas

Only one study used the psoas, a paraspinal muscle positioned in the lower lumbar region of the spine that extends through the pelvis to the femur, as the outcome measure for sarcopenia assessment. Yoshimura and colleagues [68] found that a low psoas muscle index (PMI) was associated with reduced disease-specific survival. While the optimal method to determine full-body SMM remains controversial, measurement of the psoas muscle has been suggested to be a simple and predictive measure of various morbidities [68, 69]. In addition, a recent study comparing the accuracy of widely used measurements for sarcopenia found that PMI was more strongly associated with 1-year survival when compared to L3 SMI [70]. Others suggest psoas muscle measurements are faster and simpler if the alternative is peripheral abdominal SMM measurement given the likelihood of ascites and abdominal wall edema that may reduce the accuracy of the latter [71]. Nevertheless, some researchers suggest that psoas muscle measurement may not be representative of overall, total body SMM considering that it is a minor muscle [72, 73]. Furthermore, because delineation of the psoas muscle requires CT imaging of the lumbar region, its application in HNC is limited.

### Cut-off values

Although CT imaging is considered to be the gold standard for non-invasive measurement of SMM and has been shown to provide practical and precise measures of body composition, the EWGSOP has stated that “… cut-off points for low muscle mass are not yet well defined for these measurements” [4, p.20]. Consequently, low muscle quantity measured in this manner is primarily used in research rather than in clinical practice [4]. This concern is highlighted in the current scoping review, as we revealed substantial heterogeneity in the selection of cut-off values used to diagnose sarcopenia in HNC. While some methods for determining cut-offs are more prevalent than others (e.g., ROC analysis, use of previously published values), the variability in assessment thresholds for sarcopenia is concerning. Wendrich et al. [36] utilized a non-gender specific cut-off value of <43.2 cm^2^/m^2^, a value which was determined by the likelihood of developing chemotherapy dose-limiting toxicity in those undergoing HNC treatment. An important consideration and concern for this approach is that it may identify a substantial number of individuals as sarcopenic. For example, Zwart and colleagues [6] found that upon using <43.2 cm^2^/m^2^ as a cut-off value, 97% of female patients were defined as sarcopenic. This may be considered a limitation of using this non-gender specific threshold. Van Rijn-Dekker et al. [11] used a gender-specific SMI threshold of <42.4 cm^2^/m^2^ in men and <30.6 cm^2^/m^2^ in women, which corresponded with the lowest gender-specific quartile. Prado et al. [35] used a statistical analysis known as optimum stratification to determine their gender specific SMI cut-off values for sarcopenia, with an SMI of <52.4 cm^2^/m^2^ for men and <38.5 cm^2^/m^2^ for women used to classify patients as sarcopenic.

The current state of inconsistency in the application of cut-off values to identify sarcopenia in HNC presents challenges in terms of understanding the true impact of this condition and making comparisons between studies. These concerns are evident in the most recent meta-analysis on sarcopenia in HNC, in which Findlay and colleagues [74] noted that a lack of consistency in SMI threshold values limited their ability to compare results from a large number of articles (e.g., only 7 were included for analysis) and called for a consensus on sarcopenia definition and assessment. The application of consistent threshold values to identify sarcopenia in HNC may also be limited by the unique characteristics of each patient group. For example, Yoshimiri et al. [68] proposed that using identical cut-off values in both Western and Asian populations may be inappropriate considering variations in body size, lifestyle, and ethnicity. Cut-off values will also differ based on what instrument is used to assess sarcopenia and are dependent on the site (i.e., vertebral level) at which skeletal muscle is measured, making it difficult to determine which is most appropriate and clinically relevant in HNC.

The use of ROC curve analysis to determine optimal cut-off values may be preferred given the effectiveness and accuracy of this method and its ability to discriminate between patients [75]. Using this approach also ensures that low SMM is measured according to the individuals being investigated and in relation to the outcome of interest. Another option would be to simply avoid using cut-offs and to perform analyses with SMM as a continuous variable. While this approach may circumvent the inherent limitations and inconsistencies associated with using cut-off values, it also may prevent the calculation of prevalence estimates, interfere with comparisons across different studies and populations, and subsequently limit the general applicability of results in a clinical context. Accordingly, it may be difficult to form a consensus and develop guidelines for research and clinical practice. More evidence is required before a consistent threshold can be applied to identify sarcopenia in HNC, and such evidence should be personalized to the characteristics of the population of interest.

### Timing of assessment

The point in time at which sarcopenia is assessed provides a clear impression of its utility in clinical care. With the majority of studies only having assessed sarcopenia prior to treatment, it is clear that current research is aimed at exploring the prognostic value of SMM loss and determining its ability to predict adverse outcomes in HNC. The assessment of sarcopenia both during and after treatment, however, could be beneficial in terms of establishing a clear pattern of loss and determining when patients would most benefit from proactive intervention. Only 4 (5%) and 21 (28%) studies assessed sarcopenia during and after treatment, respectively, highlighting a significant gap in the literature.

An important consideration with respect to the timing of measurement is the interval between assessment and beginning of treatment. Although most studies included in this review performed sarcopenia assessments prior to treatment, the timing fluctuated greatly ranging from one day to one year before treatment. Sarcopenia represents a modifiable risk factor that could potentially be targeted to improve patient outcomes. However, it remains to be seen whether there is enough time to target sarcopenia in the short period of time between assessment and the commencement of treatment. For example, in HNC, the time period between diagnosis and surgery can be up to four weeks; for CRT, this period is even shorter and should be no longer than two weeks [76]. The pre-treatment period in which muscle mass can be increased may be even more limited for some patients, such as those requiring immediate resection for oral cancer [77]. Nevertheless, a randomized trial in patients with lung cancer reported less severe postoperative complications and a significant decrease in hospital stay post-treatment in those who had undergone one week of endurance and resistance training prior to surgery [78], suggesting that even when performed over a short period of time, physical activity may reduce the negative impact of sarcopenia.

In HNC, studies investigating exercise and nutritional support interventions have demonstrated that they are feasible and report high patient satisfaction [79, 80]. Yamaguchi and colleagues [77] recently incorporated a preoperative exercise and nutritional intervention program for oral cancer patients with reduced daily activity. Although they found that a program consisting of warming up, walking, and resistance training performed 3–5 days per week for 4–6 weeks until admission for surgery was feasible, prospective follow-up is currently underway to determine the impact on postoperative complications and survival [77]. With respect to patient outcomes, approximately 8 weeks of moderate physical activity was reported to be adequate to improve muscle mass and health-related QoL in patients with liver cirrhosis [81]. In addition, Yamamoto and colleagues [82] explored the utility of pre-treatment exercise and nutritional support for older patients with gastric cancer diagnosed with sarcopenia and reported that a 1–4-week intervention significantly improved skeletal muscle strength and volume. Collectively, these results suggest that pre-treatment exercise and nutritional support is feasible and may facilitate an increase in SMM even when implemented over a short period of time. More high-quality research with larger samples is needed to explore the benefit of such intervention in reducing SMM loss and improving HNC outcomes. In addition, if sarcopenia is to be assessed during and after treatment, more clarity and consistency must be applied in terms of the relationship between its linguistic and operational definition.

Sarcopenia is often described as an age-related and progressive disorder [4], implying that its development takes time and occurs naturally. If SMM is being measured during and/or after treatment, consideration must be given to the fact that this treatment will likely have a negative impact on one’s ability to engage in physical activity, consume adequate oral intake, and, consequently, maintain muscle mass. This distinction is important because if treatment is inducing SMM loss and either causing or accelerating its development, then the “age-related” and “progressive” elements of sarcopenia may not apply. It may be more accurate to refer to such measurement as SMM loss rather than sarcopenia. Further, given that HNC is often diagnosed when tumour progression is advanced, even pre-treatment assessment of sarcopenia may not necessarily be age-related. ‘Secondary’ sarcopenia occurs in addition to aging when other variables are likely to contribute to the muscle wasting process [49]. For example, sarcopenia can occur secondary to a systemic disease that invokes an inflammatory response (e.g., organ failure and malignancy), neurological disorders, and conditions such as osteoarthritis [83]. Thus, it may be more accurate to describe sarcopenia as occurring *secondary to HNC* and its treatment. Based on these findings, future research should aim to determine the potential of early intervention to mitigate the impact of sarcopenia in HNC and whether it is possible for patients to continuously engage in physical activity during treatment.

### Limitations

While important findings have emerged from this scoping review, several limitations should be noted. First, because this was a scoping review, the quality of included studies and extracted data were not appraised prior to inclusion. The heterogeneity in methodologies, objectives, and assessment methods of the included studies can also be considered a limitation, especially considering that not all studies included a detailed description of how sarcopenia was measured and assessed. Additionally, there was significant overlap between some of the collected data (e.g., studies evaluated the impact of sarcopenia with respect to multiple different outcomes); as such, it was difficult to summarize and interpret the results. Finally, our review collected information pertaining to the assessment of other concepts that were measured in a similar manner to sarcopenia (i.e., cachexia). More information regarding the differences between these concepts and their assessment would be useful for future study designs.

Compared to those with other cancers, individuals with HNC are at a higher risk of malnutrition and muscle atrophy [84]. Thus, sarcopenia represents a critical index for those undergoing HNC treatment. Given the well-documented prognostic impact of sarcopenia [20, 21] and the fact that diminished muscle mass can be a correctable factor [49], the importance of accurate and clinically meaningful pre-treatment assessment is evident. If early assessment is performed and those at risk are identified, management strategies including nutritional counselling (e.g., the implementation of a diet regimen high in protein and consisting of high-quality amino acids) and physical activity intervention (e.g., both aerobic and resistance exercise) may be carried out in an attempt to avoid the potentially devastating complications associated with sarcopenia. Such endeavors may result in an improvement to the patient’s response to cancer treatment and overall QoL [85].

### Conclusions

The body of research on sarcopenia in HNC has grown substantially since 2016. However, given the heterogeneity in the definition of sarcopenia, the techniques and instruments used in its measurement, and the cut-off values applied to detect its presence, the optimal approach to assessment remains undetermined. At present, the most effective strategy in assessing sarcopenia may be dependent upon numerous factors, including access to resources, characteristics of the patient population and their treatment, and the potential accuracy of outcomes used to assess sarcopenia. For those with HNC, SMM measured at the level of C3 on head and neck CT imaging may represent a feasible, cost-effective, accurate, meaningful, and quick biomarker for the detection of sarcopenia. Further combining these SMM measurements with quick and reliable measures of muscle strength (i.e., handgrip strength) and/or physical performance may serve to increase the accuracy of sarcopenia assessments in those with HNC. More high-quality evidence is needed to determine the significance of SMM measured at C3 in relation to functional outcomes and QoL prior to its use in clinical settings.

## Supporting information

S1 Checklist Preferred Reporting Items for Systematic reviews and Meta-Analyses extension for Scoping Reviews (PRISMA-ScR) checklist. (DOCX)

S1 Appendix Search strategy for MEDLINE. (DOCX)

S2 Appendix Data extraction form. (DOCX)

S3 Appendix Characteristics of evidence source and concept details summary. (DOCX)

## References

[1] Jackson W, Alexander N, Schipper M, Fig L, Feng F, Jolly S. Characterization of changes in total body composition for patients with head and neck cancer undergoing chemoradiotherapy using dual-energy x-ray absorptiometry. Head & Neck. 2014 Sep;36(9):1356–62. doi: 10.1002/hed.23461 23970480

[2] Lønbro S, Dalgas U, Primdahl H, Johansen J, Nielsen JL, Overgaard J, et al. Lean body mass and muscle function in head and neck cancer patients and healthy individuals—results from the DAHANCA 25 study. Acta Oncol. 2013 Oct;52(7):1543–51. doi: 10.3109/0284186X.2013.822553 23964657

[3] Silver HJ, Dietrich MS, Murphy BA. Changes in body mass, energy balance, physical function, and inflammatory state in patients with locally advanced head and neck cancer treated with concurrent chemoradiation after low-dose induction chemotherapy. Head & Neck. 2007;29(10):893–900. 17405169 10.1002/hed.20607

[4] Cruz-Jentoft AJ, Bahat G, Bauer J, Boirie Y, Bruyère O, Cederholm T, et al. Sarcopenia: revised European consensus on definition and diagnosis. Age and Ageing. 2019 Jan 1;48(1):16–31. doi: 10.1093/ageing/afy169 30312372 PMC6322506

[5] von Haehling S, Morley JE, Anker SD. An overview of sarcopenia: facts and numbers on prevalence and clinical impact. J Cachexia Sarcopenia Muscle. 2010 Dec;1(2):129–33. doi: 10.1007/s13539-010-0014-2 21475695 PMC3060646

[6] Zwart AT, Hoorn A, Ooijen PMA, Steenbakkers RJHM, Bock GH, Halmos GB. CT‐measured skeletal muscle mass used to assess frailty in patients with head and neck cancer. Journal of Cachexia, Sarcopenia and Muscle. 2019 Oct;10(5):1060–9. doi: 10.1002/jcsm.12443 31134765 PMC6818448

[7] Alshadwi A, Nadershah M, Carlson ER, Young LS, Burke PA, Daley BJ. Nutritional Considerations for Head and Neck Cancer Patients: A Review of the Literature. Journal of Oral and Maxillofacial Surgery. 2013 Nov 1;71(11):1853–60. 1. doi: 10.1016/j.joms.2013.04.028 23845698

[8] Givens DJ, Karnell LH, Gupta AK, Clamon GH, Pagedar NA, Chang KE, et al. Adverse events associated with concurrent chemoradiation therapy in patients with head and neck cancer. Arch Otolaryngol Head Neck Surg. 2009 Dec;135(12):1209–17. doi: 10.1001/archoto.2009.174 20026818

[9] Marta GN, Silva V, de Andrade Carvalho H, de Arruda FF, Hanna SA, Gadia R, et al. Intensity-modulated radiation therapy for head and neck cancer: Systematic review and meta-analysis. Radiotherapy and Oncology. 2014 Jan 1;110(1):9–15. doi: 10.1016/j.radonc.2013.11.010 24332675

[10] Ganju RG, Morse R, Hoover A, TenNapel M, Lominska CE. The impact of sarcopenia on tolerance of radiation and outcome in patients with head and neck cancer receiving chemoradiation. Radiotherapy and Oncology. 2019 Aug;137:117–24. doi: 10.1016/j.radonc.2019.04.023 31085391

[11] van Rijn-Dekker MI, van den Bosch L, van den Hoek JGM, Bijl HP, van Aken ESM, van der Hoorn A, et al. Impact of sarcopenia on survival and late toxicity in head and neck cancer patients treated with radiotherapy. Radiotherapy and Oncology. 2020 Jun;147:103–10. doi: 10.1016/j.radonc.2020.03.014 32251949

[12] Karsten RT, Chargi N, van der Molen L, et al. Dysphagia, trismus and speech impairment following radiation-based treatment for advanced stage oropharyngeal carcinoma: a one-year prospective evaluation. *Eur Arch Otorhinolaryngol*. 2022;279(2):1003–1027. doi: 10.1007/s00405-021-06870-x 34043065

[13] Bril SI, Pezier TF, Tijink BM, Janssen LM, Braunius WW, Bree R. Preoperative low skeletal muscle mass as a risk factor for pharyngocutaneous fistula and decreased overall survival in patients undergoing total laryngectomy. Head & Neck. 2019 Jun;41(6):1745–55. doi: 10.1002/hed.25638 30663159 PMC6590286

[14] Grossberg AJ, Chamchod S, Fuller CD, Mohamed ASR, Heukelom J, Eichelberger H, et al. Association of Body Composition With Survival and Locoregional Control of Radiotherapy-Treated Head and Neck Squamous Cell Carcinoma. JAMA Oncol. 2016 Jun 1;2(6):782. doi: 10.1001/jamaoncol.2015.6339 26891703 PMC5080910

[15] Karsten RT, Al‐Mamgani A, Bril SI, Tjon‐A‐Joe S, Molen L, Boer JP, et al. Sarcopenia, a strong determinant for prolonged feeding tube dependency after chemoradiotherapy for head and neck cancer. Head & Neck. 2019 Nov;41(11):4000–8. 31472000 10.1002/hed.25938

[16] Cho Y, Kim JW, Lee IJ, Keum KC, Lee CG, Jeung HC. Prognostic significance of sarcopenia with inflammation in patients with head and neck cancer who underwent definitive chemoradiotherapy. Frontiers in Oncology. 2018;8(OCT):457. doi: 10.3389/fonc.2018.00457 30460194 PMC6232888

[17] Chargi N, Bril SI, Emmelot-Vonk MH, de Bree R. Sarcopenia is a prognostic factor for overall survival in elderly patients with head-and-neck cancer. Eur Arch Otorhinolaryngol. 2019;276(5):1475–86. doi: 10.1007/s00405-019-05361-4 30830300 PMC6458984

[18] Hua X, Huang X, Huang HY, Wen W, Long ZQ, Liao JF, et al. Sarcopenia is associated with higher toxicity and poor prognosis of nasopharyngeal carcinoma. Therapeutic Advances in Medical Oncology. 2020;12. doi: 10.1177/1758835920947612 32913446 PMC7444117

[19] Stone L, Olson B, Mowery A, Krasnow S, Jiang A, Li R, et al. Association Between Sarcopenia and Mortality in Patients Undergoing Surgical Excision of Head and Neck Cancer. JAMA Otolaryngology-Head & Neck Surgery. 2019;145(7):647–54. doi: 10.1001/jamaoto.2019.1185 31169874 PMC6555480

[20] Hua X, Liu S, Liao JF, Wen W, Long ZQ, Lu ZJ, et al. When the Loss Costs Too Much: A Systematic Review and Meta-Analysis of Sarcopenia in Head and Neck Cancer. Front Oncol. 2019;9:1561. doi: 10.3389/fonc.2019.01561 32117787 PMC7012991

[21] Wong A, Zhu D, Kraus D, Tham T. Radiologically Defined Sarcopenia Affects Survival in Head and Neck Cancer: A Meta-Analysis. The Laryngoscope. 2021;131(2):333–41. doi: 10.1002/lary.28616 32220072

[22] Jung AR, Roh JL, Kim JS, Kim SB, Choi SH, Nam SY, et al. Prognostic value of body composition on recurrence and survival of advanced-stage head and neck cancer. European Journal of Cancer. 2019 Jul 1;116:98–106. doi: 10.1016/j.ejca.2019.05.006 31185387

[23] MorleyJE. Sarcopenia: diagnosis and treatment. J Nutr Health Aging. 2008 Sep;12(7):452–6. doi: 10.1007/BF02982705 18615226

[24] Han A, Bokshan SL, Marcaccio SE, DePasse JM, Daniels AH. Diagnostic Criteria and Clinical Outcomes in Sarcopenia Research: A Literature Review. Journal of Clinical Medicine. 2018 Apr;7(4):70. doi: 10.3390/jcm7040070 29642478 PMC5920444

[25] Sarcopenia Keller K. Wien Med Wochenschr. 2018;169(7):157–72. 29411194 10.1007/s10354-018-0618-2

[26] Antunes AC, Araújo DA, Veríssimo MT, Amaral TF. Sarcopenia and hospitalisation costs in older adults: a cross-sectional study. Nutr Diet. 2017 Feb;74(1):46–50. doi: 10.1111/1747-0080.12287 28731551

[27] Beaudart C, Dawson A, Shaw SC, Harvey NC, Kanis JA, Binkley N, et al. Nutrition and physical activity in the prevention and treatment of sarcopenia: systematic review. Osteoporos Int. 2017 Jun 1;28(6):1817–33. doi: 10.1007/s00198-017-3980-9 28251287 PMC5457808

[28] De Buyser SL, Petrovic M, Taes YE, Toye KRC, Kaufman JM, Lapauw B, et al. Validation of the FNIH sarcopenia criteria and SOF frailty index as predictors of long-term mortality in ambulatory older men. Age Ageing. 2016 Sep;45(5):602–8. doi: 10.1093/ageing/afw071 27126327

[29] Chang KV, Hsu TH, Wu WT, Huang KC, Han DS. Association Between Sarcopenia and Cognitive Impairment: A Systematic Review and Meta-Analysis. Journal of the American Medical Directors Association. 2016 Dec 1;17(12):1164.e7–1164.e15. doi: 10.1016/j.jamda.2016.09.013 27816484

[30] Dos Santos L, Cyrino ES, Antunes M, Santos DA, Sardinha LB. Sarcopenia and physical independence in older adults: the independent and synergic role of muscle mass and muscle function. J Cachexia Sarcopenia Muscle. 2017 Apr;8(2):245–50. doi: 10.1002/jcsm.12160 27897417 PMC5377449

[31] Malmstrom TK, Miller DK, Simonsick EM, Ferrucci L, Morley JE. SARC-F: a symptom score to predict persons with sarcopenia at risk for poor functional outcomes. J Cachexia Sarcopenia Muscle. 2016 Mar;7(1):28–36. doi: 10.1002/jcsm.12048 27066316 PMC4799853

[32] Arksey H, O’Malley L. Scoping studies: towards a methodological framework. International Journal of Social Research Methodology. 2005 Feb 1;8(1):19–32.

[33] Peters M, Godfrey C, McInerney P, Munn Z, Trico A, Khalil H. Chapter 11: Scoping Reviews. In: Aromataris E, Munn Z, editors. JBI Manual for Evidence Synthesis [Internet]. JBI; 2020 [cited 2021 Jun 7]. Available from: https://wiki.jbi.global/display/MANUAL/Chapter+11%3A+Scoping+reviews

[34] Briscoe S, Bethel A, Rogers M. Conduct and reporting of citation searching in Cochrane systematic reviews: A cross-sectional study. Research Synthesis Methods. 2020;11(2):169–80. doi: 10.1002/jrsm.1355 31127978 PMC7079050

[35] Prado CMM, Lieffers JR, McCargar LJ, Reiman T, Sawyer MB, Martin L, et al. Prevalence and clinical implications of sarcopenic obesity in patients with solid tumours of the respiratory and gastrointestinal tracts: a population-based study. The Lancet Oncology. 2008 Jul;9(7):629–35. doi: 10.1016/S1470-2045(08)70153-0 18539529

[36] Wendrich AW, Swartz JE, Bril SI, Wegner I, de Graeff A, Smid EJ, et al. Low skeletal muscle mass is a predictive factor for chemotherapy dose-limiting toxicity in patients with locally advanced head and neck cancer. Oral Oncology. 2017 Aug 1;71:26–33. doi: 10.1016/j.oraloncology.2017.05.012 28688687

[37] Meerkerk CDA, Chargi N, de Bree R, de Jong PA, van den Bos F. Sarcopenia measured with handgrip strength and skeletal muscle mass to assess frailty in older patients with head and neck cancer. Journal of Geriatric Oncology. 2021;12(3):434–40. doi: 10.1016/j.jgo.2020.10.002 33067163

[38] Kagifuku Y, Tohara H, Wakasugi Y, Susa C, Nakane A, Minakuchi S, et al. What factors affect changes in body composition and swallowing function in patients hospitalized for oral cancer surgery? Clinical Interventions in Aging. 2020;15:1–7. doi: 10.2147/CIA.S235170 32021128 PMC6954079

[39] Nishikawa D, Hanai N, Suzuki H, Koide Y, Beppu S, Hasegawa Y. The Impact of Skeletal Muscle Depletion on Head and Neck Squamous Cell Carcinoma. ORL. 2018;80(1):1–9. doi: 10.1159/000485515 29393251

[40] Morley JE, Abbatecola AM, Argiles JM, Baracos V, Bauer J, Bhasin S, et al. Sarcopenia with limited mobility: an international consensus. J Am Med Dir Assoc. 2011 Jul;12(6):403–9. doi: 10.1016/j.jamda.2011.04.014 21640657 PMC5100674

[41] Kera T, Kawai H, Hirano H, Kojima M, Watanabe Y, Motokawa K, et al. Limitations of SARC-F in the diagnosis of sarcopenia in community-dwelling older adults. Archives of Gerontology and Geriatrics. 2020 Mar 1;87:103959. doi: 10.1016/j.archger.2019.103959 31945638

[42] Rubbieri G, Mossello E, Di Bari M. Techniques for the diagnosis of sarcopenia. Clin Cases Miner Bone Metab. 2014;11(3):181–4. 25568650 PMC4269140

[43] Beaudart C, McCloskey E, Bruyère O, Cesari M, Rolland Y, Rizzoli R, et al. Sarcopenia in daily practice: assessment and management. BMC Geriatrics. 2016 Oct 5;16(1):170. doi: 10.1186/s12877-016-0349-4 27716195 PMC5052976

[44] Mitsiopoulos N, Baumgartner RN, Heymsfield SB, Lyons W, Gallagher D, Ross R. Cadaver validation of skeletal muscle measurement by magnetic resonance imaging and computerized tomography. Journal of Applied Physiology. 1998 Jul 1;85(1):115–22. doi: 10.1152/jappl.1998.85.1.115 9655763

[45] Mourtzakis MM, Prado CMMPMM, Lieffers JRLR, Reiman TR, McCargar LJMJ, Baracos VEBE. A practical and precise approach to quantification of body composition in cancer patients using computed tomography images acquired during routine care. Applied Physiology, Nutrition, and Metabolism [Internet]. 2008 Sep 25 [cited 2021 Apr 6]; Available from: https://cdnsciencepub.com/doi/abs/10.1139/H08-075 18923576 10.1139/H08-075

[46] Shachar SS, Williams GR, Muss HB, Nishijima TF. Prognostic value of sarcopenia in adults with solid tumours: A meta-analysis and systematic review. European Journal of Cancer (Oxford, England: 1990). 2016 Apr;57:58–67. doi: 10.1016/j.ejca.2015.12.030 26882087

[47] Swartz JE, Pothen AJ, Wegner I, Smid EJ, Swart KMA, de Bree R, et al. Feasibility of using head and neck CT imaging to assess skeletal muscle mass in head and neck cancer patients. Oral Oncology. 2016 Nov 1;62:28–33. doi: 10.1016/j.oraloncology.2016.09.006 27865369

[48] Cooper C, Fielding R, Visser M, van Loon LJ, Rolland Y, Orwoll E, et al. Tools in the assessment of sarcopenia. Calcif Tissue Int. 2013 Sep;93(3):201–10. doi: 10.1007/s00223-013-9757-z 23842964 PMC3744387

[49] Cruz-Jentoft AJ, Baeyens JP, Bauer JM, Boirie Y, Cederholm T, Landi F, et al. Sarcopenia: European consensus on definition and diagnosis: Report of the European Working Group on Sarcopenia in Older People. Age and Ageing. 2010 Jul 1;39(4):412–23. doi: 10.1093/ageing/afq034 20392703 PMC2886201

[50] Fattouh M, Chang GY, Patel VM, Ow TJ, Rosenblatt G, Prystowsky MB, et al. Association between pretreatment obesity, sarcopenia, and survival in patients with head and neck cancer. Head and Neck. 2019;41(3):707–14. doi: 10.1002/hed.25420 30582237 PMC6709588

[51] Chang SW, Hsu CM, Tsai YH, Chang GH, Tsai MS, Huang EI, et al. Prognostic Value of Third Cervical Vertebra Skeletal Muscle Index in Oral Cavity Cancer: A Retrospective Study. Laryngoscope. 2021;131(7):E2257–65. doi: 10.1002/lary.29390 33433021

[52] Bril SI, Pothen AJ, de Bree R, Wendrich AW, Swartz JE, Wegner I, et al. Interobserver agreement of skeletal muscle mass measurement on head and neck CT imaging at the level of the third cervical vertebra. European Archives of Oto-Rhino-Laryngology. 2019;276(4):1175–82. doi: 10.1007/s00405-019-05307-w 30689037 PMC6426814

[53] Almada-Correia I, Neves PM, Mäkitie A, Ravasco P. Body Composition Evaluation in Head and Neck Cancer Patients: A Review. Front Oncol. 2019;9:1112. doi: 10.3389/fonc.2019.01112 31788443 PMC6854012

[54] Ufuk F, Herek D, Yuksel D. Diagnosis of Sarcopenia in Head and Neck Computed Tomography: Cervical Muscle Mass as a Strong Indicator of Sarcopenia. Clinical and experimental otorhinolaryngology. 2019;12(3):317–24. doi: 10.21053/ceo.2018.01613 30947498 PMC6635710

[55] Robbins KT, Shaha AR, Medina JE, Califano JA, Wolf GT, Ferlito A, et al. Consensus Statement on the Classification and Terminology of Neck Dissection. Archives of Otolaryngology–Head & Neck Surgery. 2008 May 1;134(5):536–8. doi: 10.1001/archotol.134.5.536 18490577

[56] Lindberg R. Distribution of cervical lymph node metastases from squamous cell carcinoma of the upper respiratory and digestive tracts. Cancer. 1972;29(6):1446–9. doi: 10.1002/1097-0142(197206)29:6&lt;1446::aid-cncr2820290604&gt;3.0.co;2-c 5031238

[57] Yeh KY, Ling HH, Wang CH, Chang PH, Ng SH, Chou WC, et al. Role of the appendicular skeletal muscle index for predicting the recurrence-free survival of head and neck cancer. Diagnostics. 2021;11(2):309. doi: 10.3390/diagnostics11020309 33673006 PMC7918727

[58] Brown JC, Schmitz KH. Weight lifting and appendicular skeletal muscle mass among breast cancer survivors: a randomized controlled trial. Breast Cancer Res Treat. 2015 Jun;151(2):385–92. doi: 10.1007/s10549-015-3409-0 25935584 PMC4596259

[59] Nishigori T, Okabe H, Tanaka E, Tsunoda S, Hisamori S, Sakai Y. Sarcopenia as a predictor of pulmonary complications after esophagectomy for thoracic esophageal cancer. J Surg Oncol. 2016 May;113(6):678–84. doi: 10.1002/jso.24214 26936808

[60] Romanello V, Scalabrin M, Albiero M, Blaauw B, Scorrano L, Sandri M. Inhibition of the Fission Machinery Mitigates OPA1 Impairment in Adult Skeletal Muscles. Cells. 2019 Jun 15;8(6):597. doi: 10.3390/cells8060597 31208084 PMC6627087

[61] Saitoh M, Ishida J, Konishi M, Springer J. The concept that focuses on oral motor and feeding function in cancer patients with muscle wasting: Skeletal muscle mass is associated with severe dysphagia in cancer patients. J Cachexia Sarcopenia Muscle. 2016 May;7(2):233–4. doi: 10.1002/jcsm.12119 27493876 PMC4864166

[62] Hu P, Uhlich R, White J, Kerby J, Bosarge P. Sarcopenia Measured Using Masseter Area Predicts Early Mortality following Severe Traumatic Brain Injury. J Neurotrauma. 2018 Oct 15;35(20):2400–6. doi: 10.1089/neu.2017.5422 29631485

[63] Gaszynska E, Godala M, Szatko F, Gaszynski T. Masseter muscle tension, chewing ability, and selected parameters of physical fitness in elderly care home residents in Lodz, Poland. Clin Interv Aging. 2014 Jul 22;9:1197–203. doi: 10.2147/CIA.S66672 25092969 PMC4113568

[64] Yamaguchi K, Tohara H, Hara K, Nakane A, Yoshimi K, Nakagawa K, et al. Factors associated with masseter muscle quality assessed from ultrasonography in community-dwelling elderly individuals: A cross-sectional study. Arch Gerontol Geriatr. 2019 Jun;82:128–32. doi: 10.1016/j.archger.2019.02.003 30780049

[65] Hwang Y, Lee YH, Cho DH, Kim M, Lee DS, Cho HJ. Applicability of the masseter muscle as a nutritional biomarker. Medicine (Baltimore). 2020 Feb;99(6):e19069. doi: 10.1097/MD.0000000000019069 32028430 PMC7015638

[66] Chang SW, Tsai YH, Hsu CM, Huang EI, Chang GH, Tsai MS, et al. Masticatory muscle index for indicating skeletal muscle mass in patients with head and neck cancer. PLoS ONE. 2021;16(5 May 2021):e0251455. doi: 10.1371/journal.pone.0251455 33970954 PMC8109770

[67] van Heusden HC, Chargi N, Dankbaar JW, Smid EJ, de Bree R. Masseter muscle parameters can function as an alternative for skeletal muscle mass assessments on cross-sectional imaging at lumbar or cervical vertebral levels. Quant Imaging Med Surg. 2022;12(1):15–27. doi: 10.21037/qims-21-43 34993057 PMC8666780

[68] Yoshimura T, Suzuki H, Takayama H, Higashi S, Hirano Y, Tezuka M, et al. Impact of preoperative low prognostic nutritional index and high intramuscular adipose tissue content on outcomes of patients with oral squamous cell carcinoma. Cancers. 2020;12(11):1–10. doi: 10.3390/cancers12113167 33126582 PMC7692578

[69] Waki Y, Irino T, Makuuchi R, Notsu A, Kamiya S, Tanizawa Y, et al. Impact of Preoperative Skeletal Muscle Quality Measurement on Long-Term Survival After Curative Gastrectomy for Locally Advanced Gastric Cancer. World J Surg. 2019 Dec 1;43(12):3083–93. doi: 10.1007/s00268-019-05145-1 31482345

[70] Golse N, Bucur PO, Ciacio O, Pittau G, Sa Cunha A, Adam R, et al. A new definition of sarcopenia in patients with cirrhosis undergoing liver transplantation. Liver Transplantation. 2017;23(2):143–54. doi: 10.1002/lt.24671 28061014

[71] Nam NH, Kaido T, Uemoto S. Assessment and significance of sarcopenia in liver transplantation. Clin Transplant. 2019 Dec;33(12). doi: 10.1111/ctr.13741 31651060

[72] Ebadi M, Wang CW, Lai JC, Dasarathy S, Kappus MR, Dunn MA, et al. Poor performance of psoas muscle index for identification of patients with higher waitlist mortality risk in cirrhosis. Journal of Cachexia, Sarcopenia and Muscle. 2018;9(6):1053–62. doi: 10.1002/jcsm.12349 30269421 PMC6240754

[73] Rutten IJG, Ubachs J, Kruitwagen RFPM, Beets-Tan RGH, Olde Damink SWM, Van Gorp T. Psoas muscle area is not representative of total skeletal muscle area in the assessment of sarcopenia in ovarian cancer. Journal of Cachexia, Sarcopenia and Muscle. 2017;8(4):630–8. doi: 10.1002/jcsm.12180 28513088 PMC5566632

[74] Findlay M, White K, Stapleton N, Bauer J. Is sarcopenia a predictor of prognosis for patients undergoing radiotherapy for head and neck cancer? A meta-analysis. Clin Nutr. 2021;40(4):1711–8. doi: 10.1016/j.clnu.2020.09.017 32994071

[75] Hajian-Tilaki K. Receiver Operating Characteristic (ROC) Curve Analysis for Medical Diagnostic Test Evaluation. Caspian J Intern Med. 2013;4(2):627–35. 24009950 PMC3755824

[76] Gilbert R, Devries-Aboud M, Winquist E, Waldron J, McQuestion M. The Management of Head and Neck Cancer in Ontario. 2009;71.

[77] Yamaguchi T, Makiguchi T, Nakamura H, Yamatsu Y, Hirai Y, Shoda K, et al. Impact of muscle volume loss on acute oral mucositis in patients undergoing concurrent chemoradiotherapy after oral cancer resection. Int J Oral Maxillofac Surg. 2021 Sep;50(9):1195–202. doi: 10.1016/j.ijom.2020.12.005 33414037

[78] Huang J, Lai Y, Zhou X, Li S, Su J, Yang M, et al. Short-term high-intensity rehabilitation in radically treated lung cancer: a three-armed randomized controlled trial. J Thorac Dis. 2017 Jul;9(7):1919–29. doi: 10.21037/jtd.2017.06.15 28839990 PMC5542945

[79] Brown TE, Banks MD, Hughes BGM, Lin CY, Kenny LM, Bauer JD. Randomised controlled trial of early prophylactic feeding vs standard care in patients with head and neck cancer. Br J Cancer. 2017 Jun 27;117(1):15–24. doi: 10.1038/bjc.2017.138 28535154 PMC5520203

[80] Sandmael JA, Bye A, Solheim TS, Stene GB, Thorsen L, Kaasa S, et al. Feasibility and preliminary effects of resistance training and nutritional supplements during versus after radiotherapy in patients with head and neck cancer: A pilot randomized trial. Cancer. 2017 Nov 15;123(22):4440–8. doi: 10.1002/cncr.30901 28759113

[81] Zenith L, Meena N, Ramadi A, Yavari M, Harvey A, Carbonneau M, et al. Eight Weeks of Exercise Training Increases Aerobic Capacity and Muscle Mass and Reduces Fatigue in Patients With Cirrhosis. Clinical Gastroenterology and Hepatology. 2014 Nov 1;12(11):1920–1926.e2. doi: 10.1016/j.cgh.2014.04.016 24768811

[82] Yamamoto K, Nagatsuma Y, Fukuda Y, Hirao M, Nishikawa K, Miyamoto A, et al. Effectiveness of a preoperative exercise and nutritional support program for elderly sarcopenic patients with gastric cancer. Gastric Cancer. 2017 Sep 1;20(5):913–8. doi: 10.1007/s10120-016-0683-4 28032232

[83] Mijnarends DM, Koster A, Schols JMGA, Meijers JMM, Halfens RJG, Gudnason V, et al. Physical activity and incidence of sarcopenia: the population-based AGES-Reykjavik Study. Age Ageing. 2016 Sep;45(5):614–20. doi: 10.1093/ageing/afw090 27189729 PMC5027639

[84] Pressoir M, Desné S, Berchery D, Rossignol G, Poiree B, Meslier M, et al. Prevalence, risk factors and clinical implications of malnutrition in French Comprehensive Cancer Centres. Br J Cancer. 2010 Mar;102(6):966–71. doi: 10.1038/sj.bjc.6605578 20160725 PMC2844030

[85] Denison HJ, Cooper C, Sayer AA, Robinson SM. Prevention and optimal management of sarcopenia: a review of combined exercise and nutrition interventions to improve muscle outcomes in older people. CIA. 2015 May 11;10:859–69. doi: 10.2147/CIA.S55842 25999704 PMC4435046

