# Supplementary material for: Sarcopenia in head and neck cancer: A scoping review
Source: PLoS One. 2022 Nov 28;17(11):e0278135. doi: 10.1371/journal.pone.0278135 (PMC9704631; doi:10.1371/journal.pone.0278135)
Supplement: S1 Appendix — (DOCX) [file pone.0278135.s002.docx]

**Search conducted on 07/22/2021**

**Sarcopenia**

1 Sarcopenia.tw. or Sarcopenia/ or Muscular Atrophy/ or Muscle, Skeletal/

2 Muscular Atrophy.tw.

3 skeletal muscle.tw.

4 muscle mass.tw.

5 skeletal muscle index.tw.

6 1 or 2 or 3 or 4 or 5

**Head and Neck Cancer**

7 "head and neck neoplasms".mp. or exp "Head and Neck Neoplasms"/

8 "head and neck squamous cell carcinoma".tw.

9 7 or 8

10 6 and 9
