# Supplementary material for: Sarcopenia in head and neck cancer: A scoping review
Source: PLoS One. 2022 Nov 28;17(11):e0278135. doi: 10.1371/journal.pone.0278135 (PMC9704631; doi:10.1371/journal.pone.0278135)
Supplement: S2 Appendix — (DOCX) [file pone.0278135.s003.docx]

| **Evidence Source Details and Characteristics** | | | |
| --- | --- | --- | --- |
| Article ID | | |  |
| Title | | |  |
| Author(s) | | |  |
| Year of publication | | |  |
| Country of origin | | |  |
| Aims/purpose | | |  |
| Outcome measures | | |  |
| Participant Details | Sample size | |  |
|  | Age | |  |
|  | Sex (% of sample) | |  |
|  | Cancer subsite | |  |
|  | Cancer type (e.g., primary, recurrent, or metastatic.) | |  |
|  | Clinical or pathological staging (% of sample) | |  |
|  | Treatment type | |  |
|  | Patients with sarcopenia (% of sample) | |  |
| Study design | | |  |
| Summary findings | | |  |
| **Concept Details Extracted from Source of Evidence** | | | |
| Sarcopenia definition | | |  |
| Sarcopenia cut-off value(s) and reasoning | | |  |
| Measurement technique | | Measurement instrument |  |
|  |  | Location of measurement |  |
|  |  | Muscles examined |  |
|  |  | Software and individual measuring |  |
|  |  | Reliability analysis (yes/no) |  |
|  |  | Outcome (i.e., mathematical definition) |  |
| Timing of measurement | | |  |
