# Supplementary material for: Sarcopenia in head and neck cancer: A scoping review
Source: PLoS One. 2022 Nov 28;17(11):e0278135. doi: 10.1371/journal.pone.0278135 (PMC9704631; doi:10.1371/journal.pone.0278135)
Supplement: S3 Appendix — (DOCX) [file pone.0278135.s004.docx]

| **Evidence Source Details and Characteristics** | | | |
| --- | --- | --- | --- |
| Year of publication | | | 2016 (4), 2017 (4), 2018 (6), 2019 (19), 2020 (21), 2021 (22) |
| Country of origin | | | Netherlands (18), USA (14), Japan (11), Taiwan (7), China (7), South Korea – Republic of Korea (5), Canada (3), Turkey (3), France (2), Italy (2), Finland (1), Australia (1), India (1), Brazil (1) |
| Aims/purpose | | | To determine incidence/prevalence of sarcopenia (4)  To investigate optimal level of variables to diagnose sarcopenia (1)  To determine the prognostic/risk impact of sarcopenia/SMM (62)  To determine association between outcomes/intervention (e.g., treatment type) and sarcopenia/SMM (8)  To compare body composition before and after treatment (5)  To compare lean body mass loss in different body regions (1)  To assess the robustness of C3-assessed sarcopenia in terms of interobserver agreement (1)  To determine whether sarcopenia/SMM can be assessed on neck MRI (1)  To correlate SMM assessed on neck CT with abdominal imaging (1)  To correlate masticatory SMI with L3 SMI (1)  To assess the relationship between cervical PVM values and L3 SMI (1)  To compare precision error in DEXA body composition scans before and after treatment (1)  To determine if one of more height-weight formula(e) can be used clinically as a surrogate for CT-based imaging assessment of body composition before and after treatment (1)  To detail techniques for collecting, processing of CT, validating data, and data de-identification and transfer (1) |
| Outcome measures | | | **Independent:**  Sarcopenia/SMM (61)  Lean body mass (1)  Sarcopenic obesity (1)  Obesity (1)  Fat mass (2)  Fat-free mass (1)  Cachexia (2)  Weight loss (1)  Hypolbuminaemia (1)  HPV/p16 status (1)  TNM stage (1)  Plasma EBV-DNA (1)  Myostaetosis (1)  Tumour site (1)  Comorbidities (2)  Strength training intervention (1)  Preventative rehabilitation program (1)  Nutritional program (1)  Intramuscular adipose tissue content (2)  Subcutaneous adipose tissue (2)  Visceral adipose tissue (1)  Three common lean body mass (LBM) formulae (1)  Nutritional status (5)  Dietary intake (1)  Systemic inflammation (3)  Monocyte to lymphocyte ratio -MLR (1)  Dysphagia (1)  Treatment type (2)  **Dependent:**  **Body composition (13)**  **QoL (1)**  **Nutrition-related (3)**  Intervention feasibility (1)  Locoregional control (1)  Biochemical markers (1)  Inflammation (2)  Postoperative delirium (1)  **Complications and treatment-related toxicity (29)**  **Interruptions to treatment (3)**  Length of hospital stay (1)  Unplanned hospital admissions (1)  Healthcare costs (1)  Blood transfusions (1)  Frailty (4)  Discharge disposition (1)  Inter-observer agreement of C3-assessed sarcopenia (1)  Precision error in DEXA composition scans (1)  Correlation between C3 and L3 SMM (1)  Correlation between masticatory SMI and L3 SMI (1)  Correlation between PVM and SCM areas at C2, C3, and C4 (1)  **Survival (68)** |
| Participant Details | Sample size | | 19, 22, 26, 26, 28, 30, 34, 41, 41, 45, 47, 48, 52, 52, 54, 54, 60, 60, 65, 70, 71, 73, 78, 79, 79, 82, 85, 91, 92, 98, 103, 106, 108, 111, 112, 112, 113, 122, 122, 125, 125, 128, 150, 159, 159, 164, 168, 174, 190, 190, 206, 213, 215, 215, 216, 221, 235, 235, 239, 243, 245, 246, 258, 260, 276, 287, 305, 352, 394, 441, 744, 802, 806, 862, 881, 1767 |
|  | Age (years)  [Mean (SD) or median (range)] | | 45 (18-84), 45 (18-84), 45 (18-84), 45.7 (23-69), 46 (18-79), 51 (45-58), 51.4 (11.39), 52 (9), 52.67 (14.13), 53.4 (8.3), 54.5 (9.4), 55 (12), 55.4 (1.5), 55.9 (8.8), 56.5 (0.2), 56.8 (7.3), 57.21 (9.78), 57.8 (10.8), 57.9 (10.3), 58.5 (12.8), 58.6 (8), 59 (7), 59 (18-94), 59.37 (8.4), 59.4 (13), 59.6 (13.9), 59.66 (9.12), 60, 60 (10), 60 (12), 60, 19-88), 60.3 (10.8), 60.3 (11.2), 60.3 (11.2), 60.4 (11.7), 60.4 (13.7), 61, 61 (11.6), 61 (61-64), 61.1 (11), 61.13 (9.04), 61.7 (10.9), 61.9 (10.5), 62.17 (7.22), 62.2 (12.1), 62.3 (7.8), 62.3 (10), 63 (9), 63 (39-81), 63.6 (9.6), 64 (33-70), 64 (56-73), 64.2 (8.3), 62.4 (10.2), 63.2, 63.5 (12.91), 64 (10), 64 (56-73), 64.7 (9.1), 65, 65 (43-85), 65 (58-74), 66.5 (45-81), 66.51 (12.5), 66.8 (11.6), 68 (59-77), 68 (60-76), 70.3 (7.26), 71.9 (5.1), 72 (41-92), 72.4 (7.1), 73.2, 81.5 (6.5), 81.73 (6.24)  N/A (2) |
|  | Sex (male, %) | | 44.7, 45, 51.1, 53.5, 58, 59, 62.3, 64, 64.3, 64.9, 65.2, 66.2, 66.7, 66.9, 67, 67, 67.2, 67.2, 68, 68, 68.2, 69.2, 69.8, 70, 70, 71, 71.7, 72, 72.5, 73.2, 74.2, 74.2, 74.6, 74.6, 74.7, 75.6, 76, 76.9, 77, 77, 78, 78.2, 80.9, 80.9, 81, 81.4, 81.5, 82, 82.1, 82.1, 82.3, 82.3, 82.7, 82.9, 83, 84., 85.3, 85.5, 85.6, 86.4, 87.2, 87.2, 89, 90.4, 90.7, 91.4, 92.3, 92.4, 96.9, 100, 100, 100, 100  N/A (2)  Total Males = 11628 (77%)  Total Females = 3466 (23%)  Male to Female Ratio = 5814:1733 = 3.35:1 |
|  | Cancer subsite  Majority? | | Oropharynx (28), Oral cavity (21), Nasopharynx (10), Larynx (9), Aerodigestive (oral cavity, oropharynx, hypopharynx, and/or larynx) (4), Pharynx (3), Hypopharynx (2) |
|  | Cancer type (e.g., primary, recurrent, or metastatic.) | | Primary (67)  Primary and recurrent (5)  Primary, recurrent, and second primary (2)  Primary and persistence/relapse (1)  Relapse (1)  Second primary (1) |
|  | Clinical or pathological staging  Majority? | | **TNM stage**  I (2), I and II (1), II (1), II and IV (3), III (8), III-IV (7), IV (33), IVa (5), IVb (1), N/A (1) |
|  | Treatment type  Majority? | | CRT (26), Surgery (21), Surgery and CRT (10), RT (9), Surgery and RT (2), Surgery with prior (C)RT (1), N/A (7) |
|  | Patients with sarcopenia (% of sample) | | 3.8, 6.6, 7.7, 12.4, 13.9, 14, 18.3, 19.7, 22.5, 24.4, 24.6, 25.4, 25.4, 25.9, 26.8, 28, 28.3, 31.5, 32.1, 32.8, 32.9, 33.3, 33.7, 35.1, 35.3, 36.7, 38.3, 38.4, 38.4, 38.7, 42, 45, 45.1, 46, 46.4, 48, 48.2, 48.2, 50.1, 50.3, 52.4, 53, 53.2, 54.5, 55.1, 55.4, 57, 57.7, 58.1, 61.5, 64, 64.8, 65.8, 70.6, 70.9, 74.7, 77.1, 78.7  N/A (19) |
| Study design   - Interventional (e.g., Randomized Controlled Trial) - Observational (e.g., Prospective Cohort, Retrospective Review) - Other (e.g., Meta-Analysis, Editorial/Opinion) | | | **Observational**  Retrospective review (56), Prospective, cohort (10), Prospective, longitudinal (6), Prospective, cross-sectional (1), Prospective, survey (1)  **Interventional**  Randomized controlled trial (1) |
| Summary findings (significant association vs. no association) | | | Significant association/predictor (62)  Non-significant association/predictor (3)  Excellent interobserver agreement (1)  Significant – correlation between masticatory SMI and L3 SMI (1)  Significant – C2 SMI, C3 SMI, C4 SMI, and SCM SMI values can be used as alternatives for the diagnosis of sarcopenia  Significant – SMM at C3 appears to be a good alternative to L3  Significant – high prevalence/incidence (3)  Significant - CSA measurements on CT and 1.5-T and 3-T MRI neck scans at the C3 level can be used interchangeably (1)  Non-significant – height-weight formulae with respect to CT-based measurements (1)  Non-significant – DEXA precision error did not change (1)  Body composition was significantly reduced from pre- to post-treatment (3) |
| **Concept Details Extracted from Source of Evidence** | | | |
| Sarcopenia definition | | | Loss of/low SMM (28)  Loss of/low SMM and function (4)  Loss of/low SMM, quality, and strength (1)  Loss of/low SMM, strength/function, and physical performance (10)  Loss of/low lean muscle mass (1)  Loss of/low lean body mass (1)  Progressive and generalized loss of SMM and strength (2)  Progressive and generalized loss of SMM and function (5)  Progressive loss of SMM (3)  Low SMM relative to healthy individuals (1)  Muscle-wasting condition (2)  Age-related loss of SMM (1)  Age-related loss of SMM and strength (1)  Age-related loss of SMM, strength, and physical performance (1)  Age-related, generalized, and progressive loss of SMM, muscle strength, and physical performance (1)  SMM depletion with or without fat loss (1)  Presence of low muscle quality or quantity (1)  Geriatric syndrome characterized by the age-related loss of muscle mass and/or muscle function or decreased physical status (1)  Body composition metric associated with poor oncological outcomes  N/A (12) |
| Sarcopenia cut-off value(s) | | | **SMM**  < 55cm2/m2 for males and <39cm2/m2 for females (1)  <52.4cm2/m2 for men; <38.5cm2/m2 for women (13)  <51.74 cm2/m2 for males and < 34.30 cm2/m2 for females (1)  <49cm^2^/m^2^ in men and <31 cm^2^/m^2^ in women (1)  < 42.4 cm2 /m2 (men) and < 30.6 cm2/m2 (women) (1)  < 42.4cm2/m2 for men and < 36.2 cm2 /m2 for women (1)  <41.6 cm2/m2 for males and <32.0 cm2 /m2 for females (2)  <40.8cm2/m2 for males and < 34.9cm2/m2 for females (2)  < 36.16cm2/m2 for males and < 21.02cm2/m2 in females (1)  < 36.02 cm2 /m2 for males, < 31.76 cm2 /m2 in females (2)  < 22.00 cm2/m2 for men and < 18.61 cm2/m2 for women (1)  < 21.99cm2/m2 in men and < 18.60 cm2/m2 in women (1)  < 7.26 kg/m2 for men and < 5.45 kg/m2 in women – using DEXA (1)  7.0 kg/m(2) for men and 5.7 kg/m(2) for women – using BIA (1)  Less than 605.77 cm^3^ and 445.42 cm^3^ for males and females (1)  <47.5cm2/m2 (1)  ≤ 46.6 cm^2^/m^2^ (1)  <45.2cm2/m2 (1)  < 43.2cm2 /m2 (11)  < 39.7 cm2 /m2 (1)  <36.02cm2/m2 (1)  < 20.71 cm2 /m2 (1)  <18.82cm2/m2 (1)  <12.7cm2/m2 – neck SMI (1)  <12.3cm2/m2 (1)  ≤ 0.97cm2 – rectus femoris (1)  <43cm2/m2 for underweight and normal weight males, <41cm2/m2 for overweight males and <41cm2/m2 for females in all BMI categories (1)  <52cm2/m2 for men and <38cm2/m2 for women for patients with BMI <30kg/m2, <54cm2/m2 or men and < 47cm2/m2 in women for patients with BMI >30kg/m2 (1)  <43cm2/m2 for males with BMI <25, and <53cm2/m2 for males with BMI <25, <41cm2/m2 for females regardless of BMI (2)  ≥8.87 kg/m2 for adults and ≥10.76 kg/m2 for the elderly - BIA (1)  <6.05 for males and <5.097 for females – AUC using PET-CT (1)  <6.1 kg/m2 – AUC using DEXA (1)  < 10^th^ percentile (1)  N/A – used as continuous variable (14)  N/A – investigated interobserver agreement (1)  N/A – investigated correlation between C3 and L3 (1)  N/A – used DEXA-derived lean body mass to determine precision error (1)  **Muscle Strength/Function**  *Handgrip strength*  28kg/m2 (1)  < 30kg for men and < 20kg for women (2)  <27 kg for men, and < 16kg for women (2)  <26 kg for men and <18 kg for women (1)  NA – used as continuous variable (1)  **Physical performance**  *Timed up and go test (TUG)*  Values over 10 seconds (up to 60 years), 8.1 seconds (60–69 years), 9.2 seconds (70–79 years) and 11.3 seconds (80–99 years) characterised impaired mobility (1)  *Gait speed*  <1m/s (1)  <0.8m/s (2)  N/A – used as continuous variable (1)  **Sarcopenia**  SARC-F scores ≥4 (1) |
| Sarcopenia cut-off value(s) REASONING | | | **SMM**  *Based on previously published cut-off values*  Prado et al. 2008 (11)  Go et al. 2016 – Korean patients using DEXA (1)  Van der Werf et al. 2018 (2)  Wendrich et al. 2017 (10)  Baracos et al. 2010 (1)  Makiguchi et al. 2019 (2)  Shen et al. 2004 (1)  Martin et al. 2013 (3)  Fearon et al. 2011 (1)  Zhuang et al. 2016 (2)  Mourtzakis et al. 2008 (1)  Jansen et al. 1985 (1)  Based on which values performed the best discriminatory ability (1)  Based on lowest gender-specific quartile (2)  Based on the EWGSOP and Foundation for the National Institute of Health criterion (1)  Based on AWGS algorithm (1)  Based on presence of chemotherapy dose-limiting toxicity – lowest log-likelihood value (2)  Based on median values (1)  Based on values below the 10^th^ percentile (1)  Optimal stratification (1)  ROC curve analysis (13)  Lowest log-likelihood (1)  **Muscle Strength/Function**  Based on the EWGSOP and Foundation for the National Institute of Health criterion (2)  Based on AWGS algorithm (1)  Cruz-Jentoft et al. 2010 (1)  Cruz-Jentoft et al. 2019 (2)  **Physical Performance**  Based on the EWGSOP and Foundation for the National Institute of Health criterion (2)  Based on AWGS algorithm (1)  Cruz-Jentoft et al. 2010 (1)  **Sarcopenia (SARC-F)**  Malstrom et al. 2016 (1) |
| Measurement technique | | Measurement instrument | **SMM**  CT (49), PET-CT (25), MRI (10), BIA (4), DEXA (4), B-mode ultrasonography (2), Harpenden skinfold caliper (2), non-stretchable tape measure (1) OMRON Karada Scan Hbf 375 Body Composition Monitor (1), N/A (1)  **Muscle Strength/Function**  Jamar hydraulic handheld dynamometer (5), Saehan® dynamometer (1), Digital dynamometer (1)  **Physical performance**  Stopwatch (5) |
|  |  | Location of SMM measurement | L3 (37), C3 (29), Full body (5), Appendicular (4 limbs)/ upper and lower extremities (3), Mid-arm circumference (2), C1 (1), C2 (1), C4 (1), T2 (1), T4 (1), Full body for DEXA (1), Quadriceps (1), Triceps skinfold (1), Rectus femoris (1) |
|  |  | Muscles examined | **Muscle quantity**  Abdominal wall musculature – transversus abdominis, external and internal obliques, rectus abdominus, psoas, erector spinae, and quadratus lumborum (17)  All muscles at L3/ entire L3 vertebral arch and transverse processes (8)  PVM and SCM (24)  PVM and SCM separately (1)  PVM only (1)  SCM only (1)  Right and left psoas muscles (1)  BIA - Lean body mass; includes muscle mass, as well as bones, bodily fluid, skin, and organs; is equal to total weight minus fat mass (2)  DEXA - Total body and regional (arm, leg, trunk, android, and gynoid) lean mass (1)  Appendicular/upper and lower extremities (arms and legs) (3)  Whole body (1)  CT - Relative muscle area (1)  Mid-arm circumference (2)  Triceps skinfold (1)  Vastus lateralis and rectus femoris (1)  Rectus femoris (1)  N/A (13)  N/A – no image contouring (1)  **Muscle quality**  Multifidus muscles (1) |
|  |  | Software | OsiriX software (3)  SmartPACS (1)  MiM Vista (1)  Maltron Bio-Scan 916-917 – BIA (1)  SECA analytics 115 – BIA (1)  SliceOmatic (20)  PACS (2)  Xelis 3D (1)  Volumetool (3)  Pinnacle (3)  enCORE (2)  ImageJ (7)  Aquarius workstation (3)  AquiriusNET Viewer (1)  Lookin’Body 120 analyser – Inbody (3)  Mirada DBx (1)  DEXA (1)  EclipseTM (1)  Ziostation2 (1)  GE Medical Systems LOGIQ E9 (1)  Worldmatch (1)  PetaVision (1)  Monaco TPS (3)  3D Slicer (1)  EV Insite Net (1)  Centricity Radiology RA1000 (1)  B-mode ultrasonography (2)  OMRON Karada Scan Hbf 375 Body Composition Monitor (1)  N/A – no software used; tape measurer and calipers (2)  N/A (8) |
|  |  | Individual measuring | **Physician**  Trained HNC surgeon with verification by a radiologist with 15 years of training in body radiology (1)  Single physician (1)  **Radiation Oncologist**  Single radiation oncologist (7)  Radiation oncologist who was blinded to treatment outcomes (1)  Single trained radiologist in two different sessions to avoid recall (1)  Single radiation oncologist - muscles delineated automatically by deep learning segmentation algorithm (1)  Single radiation oncologist and reviewed by another radiation oncologist (3)  Two independent radiologists (1)  Two independent radiologists blinded to patient outcomes (1)  **Researcher**  Single researcher (6)  Single researcher blinded for patient outcomes (6)  Single researcher supervised by an experienced head and neck radiation oncologist (1)  **Observer**  Trained observer (1)  Single observer (1)  Single observer trained in CT analysis and blinded to patient outcomes (1)  Main observer and another observer (1)  3 observers (medical student, doctoral student, and radiologist (1)  Independently by 6 observers: one experienced head and neck radiologist, one experienced head and neck radiation oncologist, and four medically trained researchers from the departments of Head and Neck Surgical Oncology, and Otorhinolaryngology Head and Neck Surgery – for interobserver agreement (1)  Uniformly trained examiners (1)  N/A (38) |
|  |  | Reliability analysis (yes/no) | No (67), Yes (5), Yes – interobserver agreement study (1)Yes – precision error study (1) |
|  |  | Outcome (i.e., mathematical definition) | **Muscle quantity**  L3 SMI (53)  C2 SMI (1)  C3 SMI (4)  C4 SMI (1)  T2 SMI (1)  T4 and L3 SMI difference (1)  SCM SMI (1)  L3 Psoas Muscle Index (1)  Appendicular SMI (3)  PVM CSA (3)  Left SCM CSA (1)  Right SCM CSA (1)  Total CSA of PVM and SCM (1)  L3 CSA (2)  CSA of vastus lateralis and rectus femoris (1)  CSA of rectus femoris (1)  SCM muscle volume in cm3 (1)  SMI using BIA (1)  Total body and regional (arm, leg, trunk, android, and gynoid) lean mass – using DEXA (1)  SMM – reported in kg using DEXA (1)  SMM – reported in kg using BIA (2)  SMM – reported in kg using OMRON body composition monitor (1)  Cervical SMM volume (1)  Mid-arm muscle area (2)  Triceps skinfold thickness (1)  **Muscle quality**  Intramuscular adipose tissue content (1)  **Muscle Strength/Function**  Handgrip strength measured in kg (7)  **Physical Performance**  Timed up and go test measured in seconds (2)  Gait speed measured in m/s (1)  Gait speed (6-minute walk test) measured in m/s (1)  Gait speed (4-minute walk test) measured in m/s (1)  **Sarcopenia (SARC-F)**  5-question self-report score (1) |
| Timing of measurement | | | **Prior to treatment**  Eve of surgery (1)  7 days (4)  7-14 days (1)  14 days (3)  21 days (1)  30 days (4)  45 days (1)  55 days (1)  60 days (2)  90 days (1)  Less than 4 weeks before treatment (1)  Less than 6 weeks before treatment (1)  Less than one month prior to treatment (4)  Less than 2 months prior to treatment (1)  Less than 3 months prior to treatment (4)  Within 6 months prior to treatment (1)  Within 1 year of treatment (1)  Moment of admission at institution (2)  Outset (beginning) of treatment (2)  Time of diagnosis (3)  Within a week of diagnosis (1)  During tumour staging (1)  During external beam radiation therapy simulation (1)  60 seconds after contrast injection (1)  N/A – only written as prior to treatment (32)  **During treatment**  Mid-treatment – day 15 ±2 (1)  1^st^, 8^th^, 15^th^, and 22^nd^ RT-fraction (1)  3^rd^ and 7^th^ week of CRT (1)  Weekly clinic visits during treatment (1)  **Post-treatment**  End of treatment/at discharge (3)  7 days after treatment (1)  8-12 weeks after treatment (1)  12 weeks after treatment (1)  1 month after treatment (3)  2 months after treatment (1)  2.77 months after treatment (1)  3 months after treatment (2)  3-6 months after treatment (1)  Within 1 year after treatment (1)  N/A – only written as after treatment (4)  Pre-intervention – 57 ± 30 days after the final RT dose (1)  Post-intervention (1) |
